# Permeably enclosed raw matrices direct analysis in real time mass spectrometry (PERM-DART-MS): a novel solution for the analysis of untreated samples

**DOI:** 10.1007/s00216-026-06692-y

**Published:** 2026-07-28

**Authors:** Alexandre Narcelli Pestana de Aguiar, Luis Miguel Gutiérrez Beleño, Francisco Radler de Aquino Neto, Gabriela Vanini

**Affiliations:** Instituto de Química, NAF – LADETEC, Universidade Federal Do Rio de Janeiro, Rio de Janeiro, RJ 21941-909 Brazil

**Keywords:** DART-MS, Untreated sample analysis, Preparation-free analysis, Raw sample analysis, Direct solid analysis, Ambient mass spectrometry

## Abstract

Direct analysis of raw matrices by Direct Analysis in Real Time mass spectrometry (DART-MS) remains limited by logistical and operational challenges, including complex handling and positioning, risks of sample degradation, and contamination of the analytical instrument. The Permeably Enclosed Raw Matrices (PERM) approach, introduced here, provides a standardized and instrument-safe strategy in which samples are confined within a semipermeable enclosure, enabling practical and reproducible analysis while preventing particulate release and moderating thermal exposure. The method was evaluated using six chemically and physically diverse matrices of forensic, toxicological, and food relevance: seized tablets, seized powders, roasted coffee, human hair, plant material, and spiked whole human blood. Across all samples, PERM-DART-HRMS delivered S/N ratios spanning four orders of magnitude (4–44 k). The approach enabled the identification of both major and low-abundance constituents — including MDMA, MDA, diazepam, lorazepam, 3-MMC, 25I-NBOMe, N-MEC, caffeine, endogenous lipids such as cholesterol, and sesquiterpenes such as dehydrocostus lactone — without extraction, solubilization, or chromatographic separation. Comparative analysis against conventional DART-HRMS workflows demonstrated effective prevention of instrumental contamination and markedly improved operational reliability and efficiency. Altogether, PERM-DART-MS provides a unified, preparation-free platform for rapid chemical screening, offering a practical and reliable alternative for multidisciplinary applications in forensic, toxicological, and food science contexts.

**Graphical Abstract:**

**Figure:**
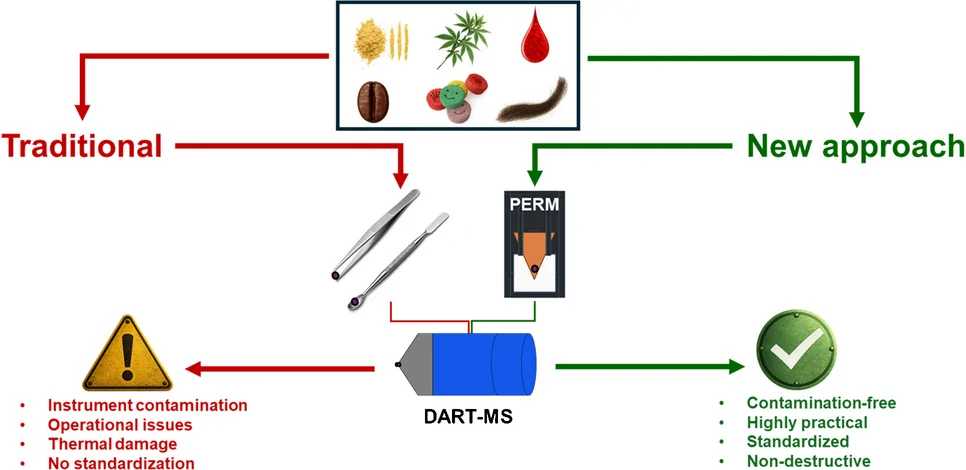

**Supplementary Information:**

The online version contains supplementary material available at https://doi.org/10.1007/s00216-026-06692-y.

## Introduction

Since its introduction by Cody et al. in 2005 [1], Direct Analysis in Real Time Mass Spectrometry (DART-MS) has increasingly established itself as a rapid and efficient analytical technology for the investigation of a wide variety of matrices and analytes [2]. DART-MS has already had a significant impact across multiple research fields, with many of its most prominent contributions occurring in forensic and food sciences [3, 4].

Despite this broad applicability, DART-MS continues to be used to a large extent for the analysis of extracts rather than raw matrices (i.e., samples in their native state, without prior extraction or preparation) [2–6]. This trend highlights a counterintuitive pattern: although the technique is widely valued for its ability to enable preparation-free analysis, most studies over the past decade have still applied DART-MS to extracts rather than directly to raw matrices. For instance, in forensic research — one of the most extensively published fields of DART-MS application — studies addressing the analysis of untreated samples remain scarce, and most date back more than 5 years [7–12].

A brief survey of the Scopus database (accessed on 14 May 2026), combining the term “direct analysis in real time” with commonly studied matrices in forensic, toxicological, and food-related contexts (e.g., “coffee”, “seized drugs”, “designer drugs”, “blood”, and “hair”), revealed that over the past 10 years (2016–2026) only one article has reported the use of DART-MS for the analysis of coffee beans [13]; three studies have analyzed seized drugs, including designer drugs, in their solid form (e.g., tablets, plant material) [7, 8, 14]; three studies have analyzed human hair [10, 15, 16], two of which were produced by the same research group and published 9 years ago; and only one article has reported the direct analysis of whole human blood without any sample preparation [17]. Extract-based DART-MS analyses of seized drugs (including designer drugs) and whole human blood account for more than twice as many published studies [18–28]. For hair and coffee, no other studies were found.

This scarcity of studies may be related to operational and technical challenges associated with the direct DART-MS analysis of matrices in their native state [7, 8, 12, 17, 20]. The few reports in the literature that mention these challenges suggest that they are associated with instrument safety and sample integrity (e.g., contamination, sample thermal damage) [7, 12], variability arising from the lack of standardization [29], and hindered analytical performance (e.g., insufficient sensitivity, carryover) [12, 17, 20]. Brown et al. [7], for example, directly introduced leaves and tablets into the DART region using tweezers and reported the risk of particulates being swept into the mass spectrometer inlet. More recently, Aguiar et al. [12] likewise reported an increased risk of contamination and carryover when analyzing unprocessed seized tablets held with tweezers in the DART-MS ionization region. Samms et al. [29] also analyzed tablets through manual introduction and reported that the position of the sample could not be standardized across analyses, making it difficult to separate the effects of sample preparation from those of sample positioning. Antonio et al. [17] in turn, analyzed raw whole blood containing benzodiazepines using a metal mesh screen as the sampling support and found that the analytes could not be detected at concentrations within the range typically found in forensic casework, leading the authors to conclude that direct DART-HRMS analysis of unextracted blood was not suitable for real-case screening without prior sample treatment. These studies, however, mentioned such challenges only briefly, and none investigated them systematically.

Although most studies within this limited literature have relied on tweezers or similar tools for the direct analysis of untreated matrices [7, 8, 12, 29], other sample-introduction strategies have been explored in specific contexts. Angled DART configurations, applied to food and plant materials, enable spatially resolved chemical profiling of exposed surfaces [30, 31]. However, they require substantial physical adaptation of the DART-MS setup, including changes to ion-source geometry, modified transfer components, customized sampling platforms, and motorized sample movement, making them specialized surface-scanning arrangements rather than broadly applicable workflows for diverse raw matrices in conventional DART-MS configurations. Thermal damage has also been reported under angled DART conditions and therefore remains a relevant concern [30, 31]. Another distinct approach is surface-residue sampling, in which residues already present on external surfaces, or collected by swab-, wipe-, or contact-based sampling, are directly analyzed [32–34]. Although useful for rapid trace screening, these workflows do not constitute analysis of the raw matrix itself: only residues present on, or transferred from, a surface under the sampling conditions are analyzed. Accordingly, the detected profile depends on residue transfer, surface accessibility, sampling material, and solvent use [32, 33], and may not reliably represent the chemical composition of the sample of interest [34].

Despite the aforementioned challenges, the analysis of untreated samples by DART-MS can offer distinct analytical advantages over extract-based workflows, including access to constituents that may be lost or whose recovery may be compromised by extraction and processing steps [12] — all within analysis times of under one minute. Conventional workflows typically require 5–30 min of instrumental analysis alone, in addition to multiple pre-treatment steps, extraction optimisation, chromatographic method development, and the use of organic solvents both for extraction and, in the case of LC–MS, for the analysis itself [35–37]. Moreover, these workflows inherently result in irreversible sample consumption. The development of strategies capable of enabling reliable DART-MS analysis of raw matrices while mitigating the associated operational and instrumental risks therefore addresses an unmet analytical demand.

In this context, the present study aimed to systematically investigate the key limitations associated with the direct analysis of raw matrices by DART-HRMS and to develop a new analytical approach based on confining samples within a semipermeable enclosure to overcome these limitations.

## Experimental

### Samples

Six types of raw matrices were analyzed, representing samples commonly encountered in forensic, food, and toxicological contexts: a seized illicit-drug tablet; two seized illicit-drug samples in powder form (one yellow and one red); roasted coffee powder; dry plant material; human hair; and antemortem human whole blood spiked with analytical standards of drugs of abuse.

All seized drug samples were provided by the Secretaria de Estado de Polícia Civil do Rio de Janeiro (SEPOL/RJ) and correspond to seizures carried out between 2022 and 2024 (SEI-SEPOL Process No.: 360007/001207/2023 and SEI-UFRJ Process No.: 23079.202338/2024–71). The roasted coffee sample consisted of a commercially available Brazilian ground coffee. The plant material consisted of a commercially purchased wormwood leaf. The hair sample consisted of natural, untreated, undyed brown human hair provided by a volunteer, in accordance with the approval granted by the Research Ethics Committee of the Faculdade de Odontologia da Universidade Federal do Rio de Janeiro (CAAE: 93056925.5.0000.0268). The antemortem blood sample was donated by the Rio de Janeiro State Institute of Hematology Arthur de Siqueira Cavalcanti, and its use was approved by the Research Ethics Committee of Clementino Fraga Filho University Hospital and the Faculty of Medicine of the Federal University of Rio de Janeiro (CAAE: 56118322.4.0000.5257).

### Analytical standards

The analytical standards used to fortify the blood sample were as follows: 7-chloro-1-methyl-5-phenyl-3H-1,4-benzodiazepin-2-one (diazepam), 7-Chloro-5-(2-chlorophenyl)−3-hydroxy-1,3-dihydro-1,4-benzodiazepin-2-one (lorazepam), 3,4-Methylenedioxymethamphetamine (MDMA), 3,4-Methylenedioxyamphetamine (MDA), 3-Methylmethcathinone (3-MMC), and 2-(4-Iodo-2,5-dimethoxyphenyl)-N-(2-methoxybenzyl)ethanamine (25I-NBOMe).

Diazepam and lorazepam were purchased from Cerilliant; 25I-NBOMe and 3-MMC from Absolute Standards Inc.; and MDMA and MDA from Sigma-Aldrich.

### Preparation of the spiked blood sample

Preparation of the spiked blood samples was performed by adding predetermined amounts of the analytical standards — in methanolic solutions (1000 µg mL⁻^1^) — to separate glass vials and allowing the solvent to evaporate completely. Whole blood was then added and homogenized by vortex mixing, yielding final analyte concentrations of 0.1 and 2.5 µg mL⁻^1^.

### Instruments and conditions

Analyses were performed using a DART ionization source (IonSense, Saugus, MA, USA) coupled to a high-resolution Q Exactive Orbitrap mass spectrometer (Thermo Fisher Scientific, Bremen, Germany). The DART source was operated in positive ionization mode with helium (99.999% purity) at 3.0 L min⁻^1^ and temperatures ranging from 200 to 350 °C. The grid electrode voltage varied between 50 and 350 V, and the distance between the DART source outlet and the HRMS inlet was set to 3 cm. The Orbitrap was operated at a resolving power of 70,000 full width at half maximum (FWHM) for full-scan experiments and 17,500 FWHM for MS^2^ experiments performed in data-dependent acquisition (DDA) mode. Normalized collision energy (NCE) was set to 20. Analysis times ranged from 13.8 to 48 s, with a mass range of *m*/*z* 100–600. The specific conditions employed for each matrix are listed in Table 1.

**Table 1:** Experimental conditions applied to each matrix

Matrix	Gas temperature (°C)	Grid voltage (V)	Analysis time (s)
Seized tablet	300	350	13.8^a^, 30
Seized powders	200	350	30
Coffee powder	200	350	30
Hair	350	50	30
Plant material	350	50	13.8^a^, 30
Blood	350	50	48

^a^Values corresponding to the extract-based workflow

### Analytical procedure

**Fig. 1:**
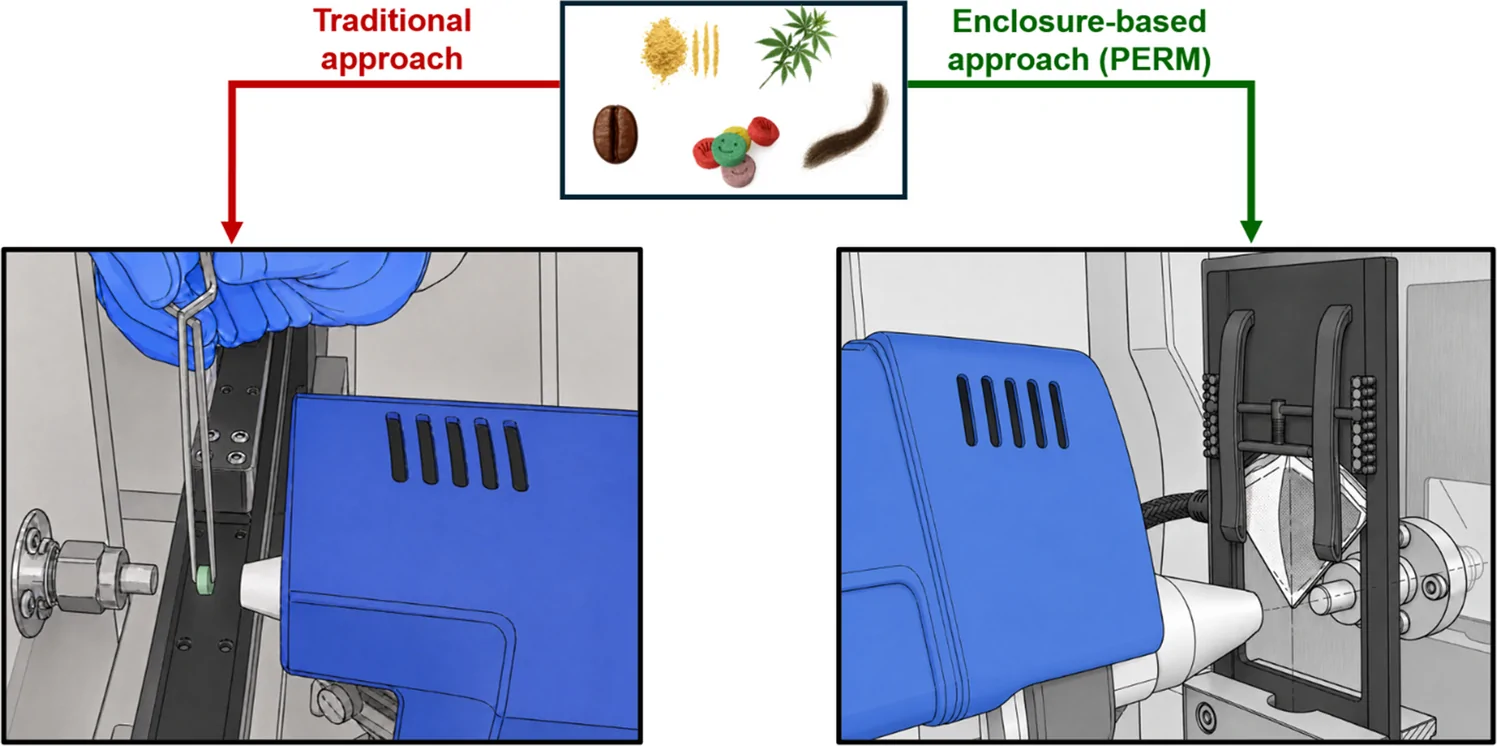
Schematic comparison between the two analytical modes evaluated: the traditional direct-analysis approach (e.g., using tweezers), in which the raw matrix is introduced directly into the ionization region, and the newly proposed enclosure-based approach

Each sample was analyzed under two main direct analysis workflows (Fig. 1): the traditional approach (i.e., using tweezers, a spatula, or a mesh screen) and the newly proposed enclosure-based approach. In addition, extract-based reference analyses were performed for the seized tablet and plant material for the focused analytical comparison described in the “Analytical comparison between PERM-DART-HRMS and reference DART-HRMS workflows” section.

In the traditional approach, approximately 20 mg of powder-type samples (seized yellow powder, seized red powder, and coffee powder) was held with a spatula between the DART source and the MS inlet, whereas the other solid samples (whole tablet, 5 mg of hair, and 10 mg of dried leaves) were held in the same region with tweezers. The blood sample was spotted (5 µL) onto a metal mesh support (OpenSpot® Bruker card), which was then positioned in the ionization region.

In the enclosure-based approach, the same amount of each sample was placed within a semipermeable enclosure prepared from a commercially available cellulose-based porous paper disk (qualitative grade; Cientec, 80 g m⁻^2^, 7 cm diameter, lot 24080312JP, manufactured in 08/2024). Each disk was manually folded with gloves into a conical enclosure by folding the circular disk in half and then folding the resulting semicircle in half. The enclosure was mechanically closed by pressure when coupled to the PLA 3D-printed holder, without adhesives, staples, or additional sealing materials. Solid samples were positioned at the vertex of the enclosure, or as close as possible to this region according to sample size and morphology, whereas the blood sample was spotted at the same vertex using an automatic pipette. The enclosure was then coupled to a custom holder device, purpose-built for this study and 3D-printed using a Creality Ender 3 v1 printer; the assembled device was subsequently integrated into the DART-HRMS setup. The holder contained a height-adjustable marker, and all experiments were carried out at the position corresponding to 54 mm from the holder base, which placed the lower region of the folded enclosure approximately along the central gas-flow axis of the ionization region. Analyses were performed in triplicate.

For comparison with extract-based DART-HRMS, extracts were prepared from the seized tablet and plant material according to the procedures used by the Civil Police of the State of Rio de Janeiro [12] and described by Nie et al. [38], respectively. For the seized tablet, 150 mg of ground tablet material was extracted with 1.0 mL of methanol by vortex mixing for 10 s; the mixture was centrifuged at 2000 rpm for 4 min, and the supernatant was diluted 500-fold with methanol. For the plant material, 5 mg of wormwood leaf material was extracted with 1.0 mL of methanol for 10 min in an ultrasonic bath. In both cases, 5 µL of the resulting extract was deposited onto a metal mesh support (OpenSpot® Bruker card) and introduced into the DART-HRMS ionization region.

## Results and discussion

The proposed approach is based on the conception and application of a system composed of a semipermeable enclosure coupled to a 3D-printed device responsible for supporting and positioning this enclosure (patent application No. BR 10 2026 004396). Although different materials may be used to construct these components — e.g., polyethylene terephthalate glycol-modified (PETG) for the device and mixed cellulose ester (MCE) or polytetrafluoroethylene (PTFE) membranes for the enclosure — the combination of 80 g m⁻^2^ porous paper for the enclosure and polylactic acid (PLA) for the 3D-printed device proved effective and cost-efficient, with material costs totaling no more than US$ 0.12 for the device and US$ 0.05 per enclosure.

**Fig. 2:**
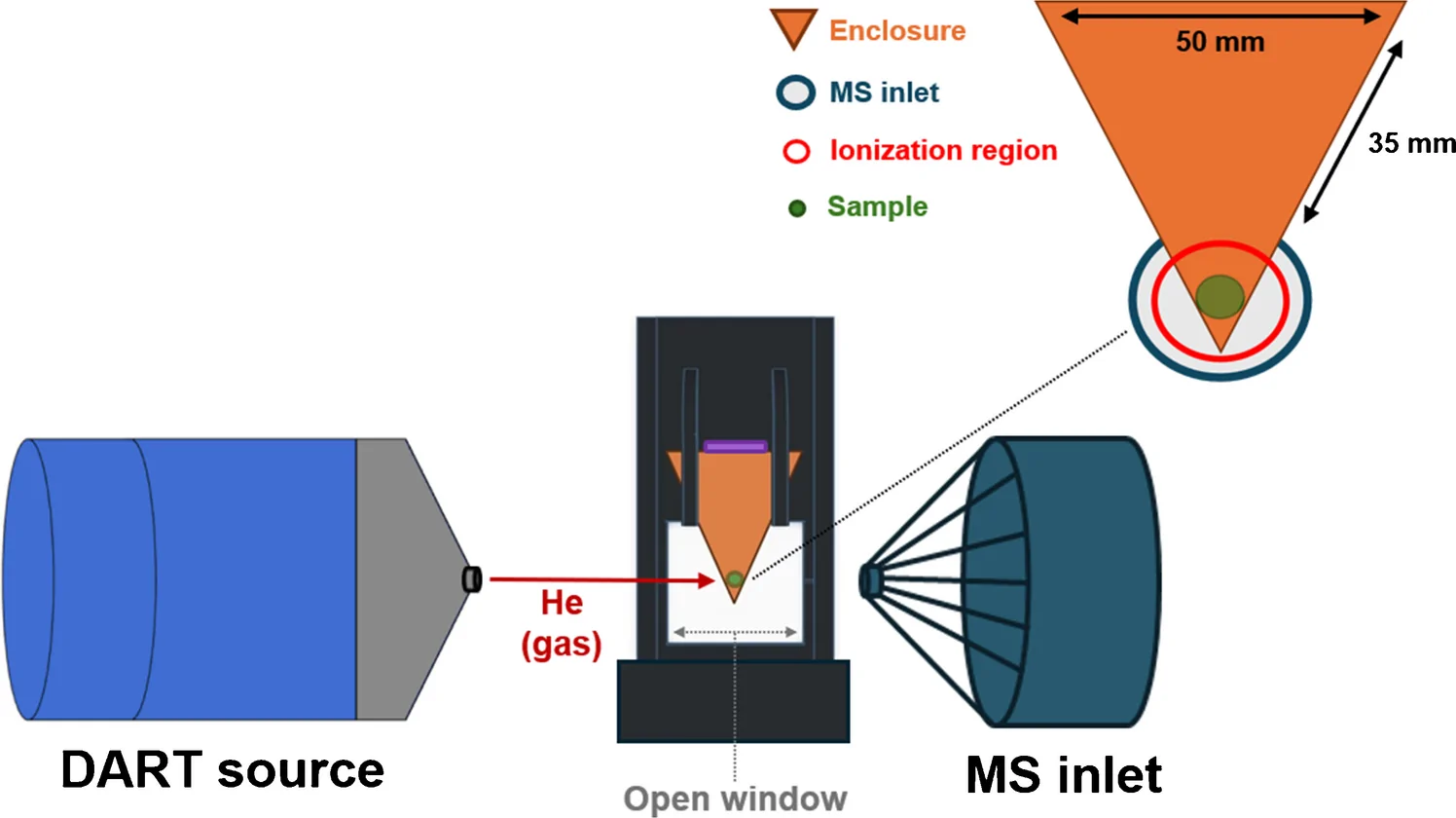
Schematic representation of the PERM assembly integrated into the DART-MS setup. The scheme shows the DART source outlet, helium gas flow through the folded enclosure, sample position, open rectangular window of the 3D-printed holder, and MS inlet

The proposed enclosure-based workflow consists of placing the sample inside the enclosure, which is then coupled to the 3D-printed device at a defined height. Once assembled, the complete unit is integrated into the DART-MS setup, as shown schematically in Figs. 1 and 2. In light of its defining characteristics and operating principle, the approach developed in this study was designated Permeably Enclosed Raw Matrices (PERM).

**Fig. 3:**
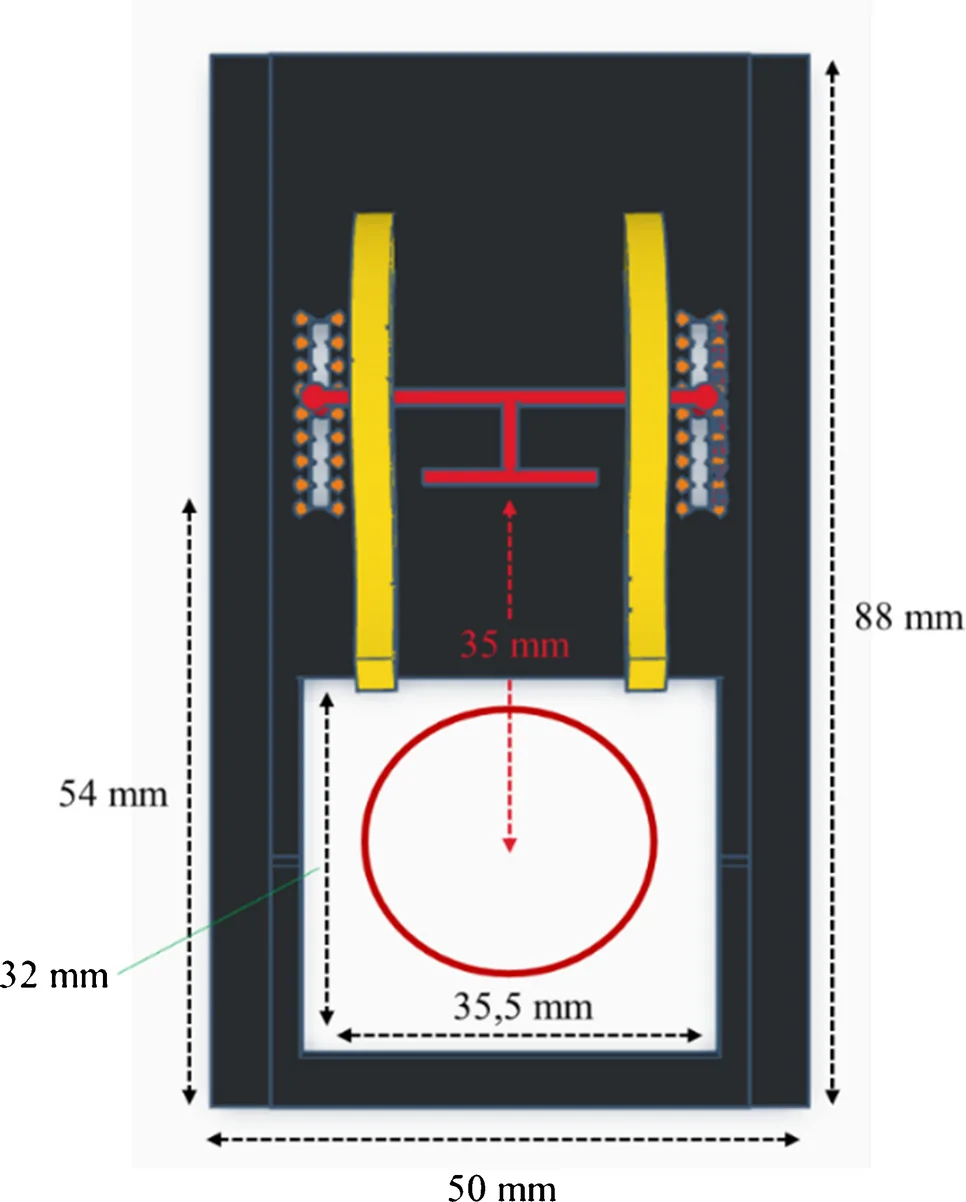
Front-view 3D-printing design of the holder used to position the folded enclosure in the DART-MS ionization region. The main device and window dimensions are indicated in millimeters. Yellow elements correspond to the pressure arms used for enclosure fixation, the red rod indicates the height adjuster, and the red circle symbolizes the ionization region within the open rectangular window

The 3D-printed holder was designed to ensure proper and standardized positioning of the enclosure, so that the specific region containing the sample is appropriately exposed to the ionization region. For conical enclosures, as employed in this study, the positioning should be as illustrated in the zoomed region of Fig. 2. To ensure this positioning, the device comprises pressure arms for mechanical fixation of the enclosure, a height-adjustable marker that acts as a mechanical stop to define the insertion level and prevent further upward displacement of the enclosure, and an open rectangular window aligned with the DART source outlet and the MS inlet (Figs. 2 and 3). This window is dimensioned to allow the heated gas stream and desorbed/ionized species to pass through the region containing the enclosed sample, while minimizing direct interaction between the ionization zone and the 3D-printed structure. As described in the “Analytical procedure” section, all experiments were performed with the enclosure fixed at the 54 mm height-marker position, which placed the lower region of the folded enclosure along the central gas-flow axis of the ionization region, corresponding to an approximate distance of 35 mm between the enclosure vertex and the height marker.

The enclosure was conceived to perform four main functions: (i) acting as a semipermeable barrier that allows desorbed ions to exit freely while retaining solid particles or fluid droplets; (ii) serving as the interface between the matrix and the heated gas stream, thereby moderating heat transfer to the sample and promoting a more controlled thermal environment; (iii) enabling more standardized and practical sampling; and (iv) automatically (i.e., non-manually) supporting the sample during analysis. In addition, samples can be transported, stored, and analyzed within the same enclosure, further enhancing practicality and saving time.

### Assessment of background contributions from the enclosure and 3D-printed holder materials

**Fig. 4:**
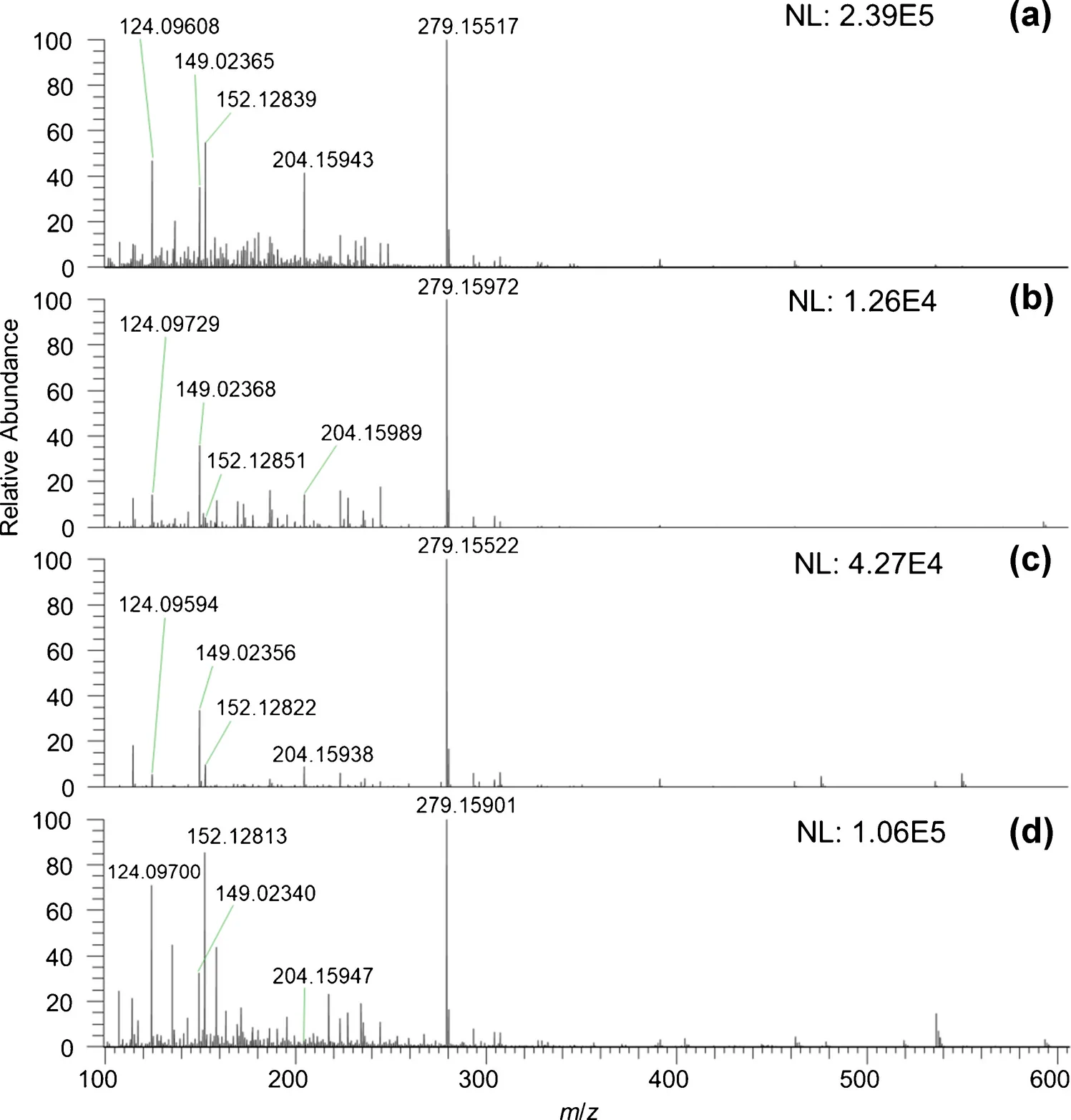
Full-scan DART-HRMS blank spectra acquired at 200 °C and 350 V under four configurations: (**a**) air/system blank, without enclosure or holder; (**b**) paper enclosure, without 3D-printed holder; (**c**) paper enclosure coupled to the PLA 3D-printed holder; and (**d**) paper enclosure coupled to the PETG 3D-printed holder. Normalized Level (N.L.) values, corresponding to signal intensity, are indicated in the spectra

**Fig. 5:**
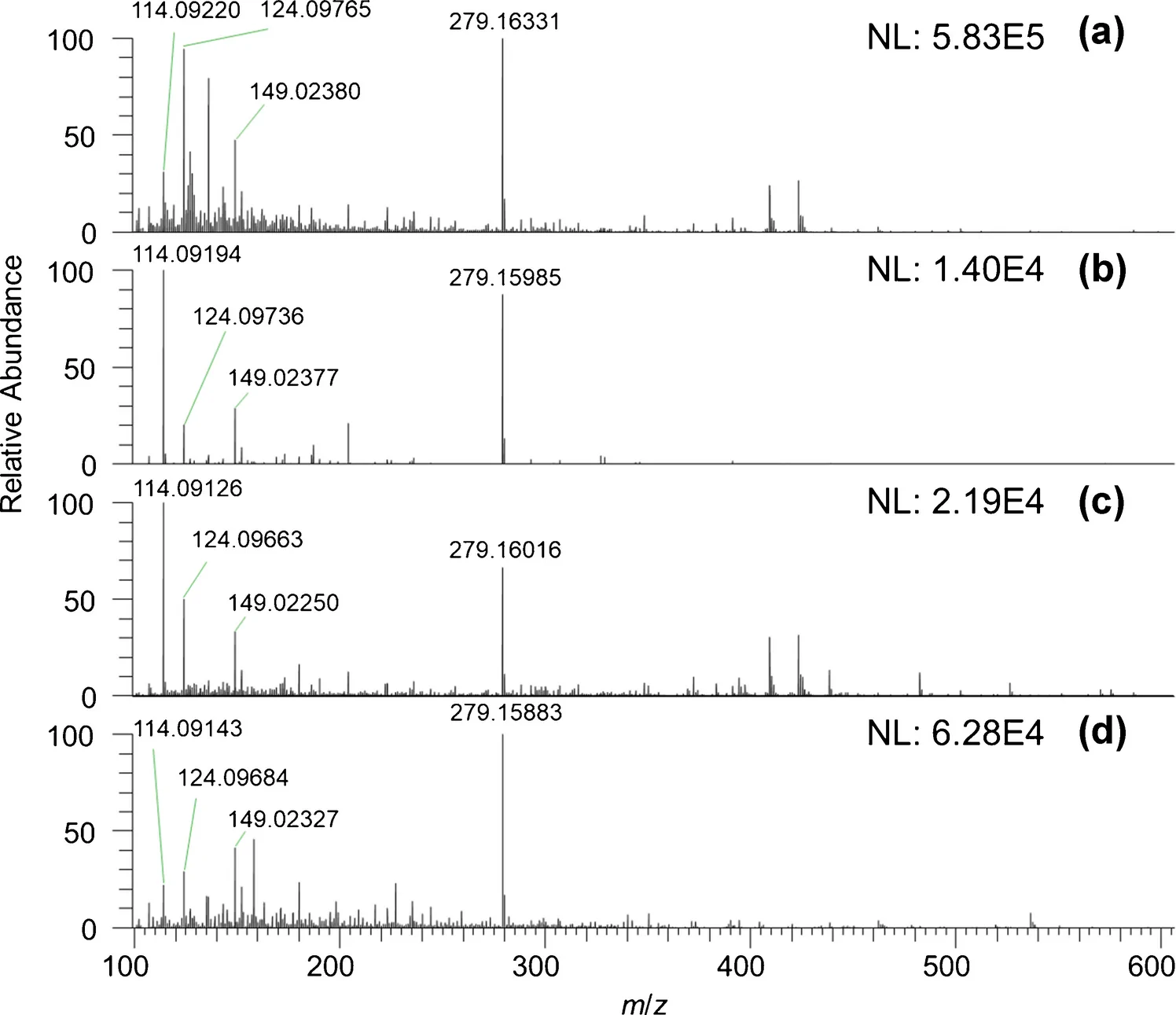
Full-scan DART-HRMS blank spectra acquired at 350 °C and 50 V under four configurations: (**a**) air/system blank, without enclosure or holder; (**b**) paper enclosure, without 3D-printed holder; (**c**) paper enclosure coupled to the PLA 3D-printed holder; and (**d**) paper enclosure coupled to the PETG 3D-printed holder. Normalized Level (N.L.) values, corresponding to signal intensity, are indicated in the spectra

Because the PERM workflow relies on a paper enclosure coupled to a 3D-printed holder, blank spectra routinely acquired before sample analyses were systematically examined to assess possible background contributions from these components. Four blank configurations were evaluated: (i) air/system blank, without enclosure or holder; (ii) paper enclosure, without 3D-printed holder; (iii) paper enclosure coupled to the PLA 3D-printed holder; and (iv) paper enclosure coupled to the PETG 3D-printed holder. The spectra obtained at 200 °C with a grid voltage of 350 V and at 350 °C with a grid voltage of 50 V are shown in Figs. 4 and 5. Together, these conditions include the settings most frequently used throughout the study and cover the lower and upper limits of gas temperature and grid voltage applied in the experiments.

Overall, the same group of background ions was observed across the four configurations under both instrumental conditions. At 200 °C and 350 V, the spectra showed background ions at *m*/*z* 124.09608, 149.02365, 152.12839, 204.15943, and 279.15517. At 350 °C and 50 V, the main background ions included *m*/*z* 114.09194, 124.09736, 149.02377, and 279.15985. These ions were already present in the air/system blanks acquired at the beginning of the corresponding blank sequences, before introduction of the enclosure/holder assemblies into the ionization region. Importantly, no new dominant ion, distinct spectral pattern, or substantial increase in background abundance was observed when the paper enclosure was analyzed alone or when it was coupled to the PLA or PETG holders.

The abundance of the background signals, measured as normalized level (N.L.), was low for the configurations containing the paper enclosure. At 200 °C and 350 V, the N.L. values were 2.39 × 10^5^ for the air/system blank, 1.26 × 10^4^ for the paper enclosure, 4.27 × 10^4^ for the paper enclosure coupled to PLA, and 1.06 × 10^5^ for the paper enclosure coupled to PETG. At 350 °C and 50 V, the corresponding values were 5.83 × 10^5^, 1.40 × 10^4^, 2.19 × 10^4^, and 6.28 × 10^4^, respectively. Thus, the blanks containing the paper enclosure, either with or without 3D-printed holders, remained within the 10^4^–10^5^ range and did not show a systematic increase under the highest gas-temperature condition, as would be expected if relevant desorbable/ionizable impurities were being released from the enclosure or holder materials.

**Fig. 6:**
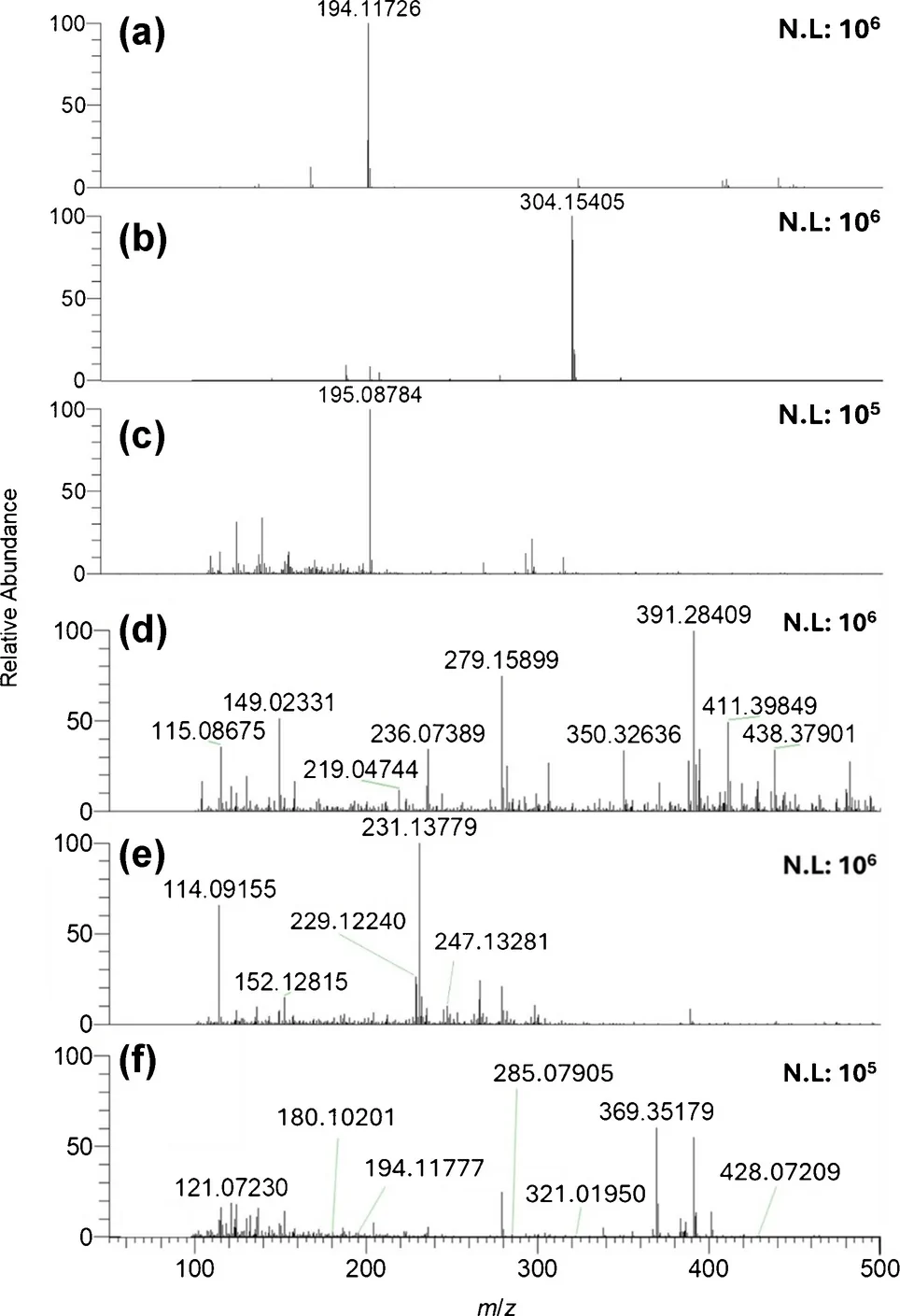
Full-scan spectra obtained by PERM-DART-HRMS for the seized tablet (**a**), seized yellow powder (**b**), roasted coffee (**c**), human hair (**d**), plant material (**e**), and spiked whole blood (**f**). Chemical structures, molecular formulas, and theoretical *m*/*z* values are provided in Table S1. Normalized Level (N.L.) values, corresponding to signal intensity, are indicated in the spectra

The comparatively higher abundance observed in the air/system blanks is consistent with the unobstructed passage of the heated gas stream through the open DART region. In this configuration, ambient and instrumental background species can be transported more freely toward the MS inlet. In contrast, the placement of the paper enclosure partially attenuates and disperses the gas stream. In the absence of sample-derived analytes, this effect resulted mainly in attenuation of the pre-existing background rather than generation of new intense ions, further indicating that the enclosure/holder assembly did not behave as a relevant source of desorbable and ionizable species under the conditions evaluated. Importantly, this attenuation did not prevent the transmission of sample-derived ions when chemically informative matrices were present inside the enclosure, as further discussed in the “Cross-matrix applicability and analytical performance of PERM-DART-HRMS” section and illustrated by the spectra obtained for the investigated matrices (Fig. 6).

The background ions observed in the blank spectra are compatible with common ambient/instrumental species in HRMS analyses. Ions at *m*/*z* 124.09608 and 152.12839 are consistent with glycol- or glycol ether-related species, which may originate from laboratory air, cleaning products, solvent vapors, ventilation systems, humidity, or residual surfactants [39]. The ions at *m*/*z* 149.02365 and 279.15985 are consistent with phthalate-related species, frequently associated with plasticizers from flexible tubing, containers, caps, gloves, laboratory surfaces, dust, ambient air, or residual inlet background [40, 41]. The ion at *m*/*z* 114.09194 is compatible with a caprolactam-like species, commonly associated with nylon/polyamide-related residues from nylon filters, membranes, fibers, packaging materials, or airborne laboratory background [42]. These assignments are consistent with the typical background profile expected in open-air ambient ionization experiments.

### Comparative assessment of operational feasibility: traditional vs. PERM mode

To assess the challenges associated with direct DART-MS analysis and evaluate the feasibility of the proposed approach in overcoming them, raw matrices representing a range of operationally demanding scenarios — including particulate, heat-sensitive, and chemically complex samples — were first investigated under the traditional approach (i.e., using tweezers, a spatula, or a mesh screen) and then under the instrumental arrangement illustrated in Figs. 1 and 2 (PERM mode).

For powder-type matrices, including roasted coffee and the two seized powder samples, analyses performed using a spatula showed that direct exposure of free-flowing particles to the heated gas stream resulted in transport of solid into the mass spectrometer, leading to interruption of the analyses. For the seized tablet held with tweezers during analysis, surface thermal damage and cracking were observed by the end of the run (Fig. S2). MDMA was detected as the major component at high absolute intensity (~ 10^8^), but a subsequent blank analysis revealed carryover of the same order of magnitude (Fig. S3). Post-analysis visual inspection further showed particulate deposition in the vapour interface module and contamination in the Orbitrap ion-transfer region in both tablet and powder experiments (Fig. S4). Handling and positioning these matrices in the ionization region also proved operationally challenging, especially for powders.

The hair sample was likewise difficult to manipulate when analyzed in a coiled configuration and held with tweezers between the DART source and the MS inlet. During positioning and analysis, instrument instability developed. Visual inspection revealed a hair strand obstructing the MS inlet orifice (Fig. S5); normal performance was restored only after its removal and local cleaning. For the plant material, direct handling with tweezers again resulted in visible contamination of the instrument, with particulate residue observed inside the vapor interface, while the sample itself exhibited signs of thermal damage.

Whole blood analyzed on a mesh support (OpenSpot® Bruker card) and subsequently introduced into the ionization region also proved problematic: sample migration toward the MS inlet was observed during analysis, and post-analysis visual inspection revealed blood contamination of the porcelain inlet component integrated into the vapour module (Fig. S6), requiring thorough cleaning.

Taken together, these attempts showed that direct DART-HRMS analysis of raw matrices by conventional sample holding or positioning in the ionization region is affected by important practical and analytical limitations. These include lack of standardization, strong dependence on operator-controlled variables, risk of sample burning or degradation, concerns related to operator exposure during sample manipulation, and, most critically, contamination of the instrument by particles or microdroplets carried into the MS by the heated gas stream.

When the same matrices were analyzed under PERM mode, none of these issues were observed (Table 2). No particulate deposition was observed in the vapour interface or ion-transfer region, the need for operator manipulation within the ionization region was effectively eliminated, and the samples showed no visible signs of thermal damage after analysis. The absence of visible thermal damage under PERM mode may be related to the reduced direct exposure of the sample to the heated helium stream. In the traditional direct-DART workflow, the sample surface is directly impacted by the hot gas flow, which can produce localized heating. Under PERM conditions, heat can still be transferred through the porous paper enclosure; however, the enclosure acts as an intermediate interface with a larger exposed surface area, which can disperse the gas flow and distribute the thermal load before heat reaches the sample-containing region. At the same time, this mediated heat transfer can still support the enhanced analyte desorption and ionization/detection expected at higher DART temperature settings, while reducing the abrupt and localized thermal input associated with direct gas-flow impingement. This interpretation should nonetheless be considered with caution, since the evidence was based on post-analysis visual inspection and no systematic study varying parameters that influence thermal input was performed. It should also be noted that, although the enclosure reduced the risk of visible thermal damage under the conditions evaluated in this study, temperatures above 400 °C or longer acquisition times (> 1 min) may substantially increase the risk of thermal damage to both the enclosed sample and the enclosure itself.

**Table 2:** Comparison between the traditional (i.e., using tweezers, a spatula, or a mesh screen) and proposed (PERM) approaches for the direct analysis of raw matrices by DART-HRMS

	Instrument contamination		Sample thermal damage		Workflow practicality and standardization	
Matrix/approach	Trad	PERM	Trad	PERM	Trad	PERM
Seized tablet	Yes	None	Observed	Not observed	Lower	Higher
Seized powders	Yes	None	n/a	n/a	Much Lower	Much Higher
Coffee powder	Yes	None	n/a	n/a	Much Lower	Much Higher
Hair	Yes	None	Not observed	Not observed	Much Lower	Much Higher
Plant material	Yes	None	Observed	Not observed	Lower	Higher
Whole blood	Yes	None	Not observed	Not observed	Similar	Similar

### Cross-matrix applicability and analytical performance of PERM-DART-HRMS

The results obtained under PERM mode are compiled in Table 3, which presents the substances identified in each matrix along with their analytical metrics. Figure 6 shows the corresponding full-scan mass spectra, with chemical structures and theoretical *m*/*z* values of the identified analytes provided in Table S1, and MS^2^ spectra in Figs. S7–S15.

**Table 3:** Substances detected in untreated matrices by PERM-DART-MS, along with their mean mass-to-charge ratios (*m*/*z*), respective mass errors (ppm), and mean signal-to-noise ratios (S/N)

Matrix	Substances	*m*/*z*	Error (ppm)	S/N
Seized tablet	MDMA	194.11742	0.94	44,385
	N-MEC	192.13847	0.89	483
Seized powders	Cocaine^a^	304.15395	1.27	43,082
	MDA^b^	180.10067	6.87	15,203
Coffee powder	Caffeine	195.08791	1.31	1015
	5-hydroxymethylfurfural	127.03929	1.81	39
	Trigonelline	138.05514	2.61	27
Hair	Squalene	411.39847	0.14	1378
	Cholesterol	369.35159^e^	0.01	121
	Di-N-octyl phthalate	391.28419	0.27	2844
	Mono-N-octyl phthalate	279.15907	0.07	2543
Plant material	Dehydrocostus lactone (M)	231.13786	0.45	13,668
	Dehydrated dehydrocostus lactone (M – H_2_O)	213.12761	1.03	46
	Dehydrogenated dehydrocostus lactone (M + O – H_2_O)	229.12244	0.55	4978
	Hydrated dehydrocostus lactone (M + H_2_O)^c^	249.14851	0.04	452
	Mono-oxygenated dehydrocostus lactone (M + O)	247.13288	0.05	657
	Mono-oxygenated dihydrated dehydrocostus lactone (M + O + 2H_2_O)	283.15397	0.11	12
Spiked blood^d^	Diazepam (2.5 µg mL⁻^1^)	285.07900	0.33	239
	Diazepam (0.1 µg mL⁻^1^)	285.07890	0.06	7
	Lorazepam	321.01946	0.82	4
	MDMA	194.11772	0.61	381
	MDA	180.10198	0.46	263
	25I-NBOMe	428.07203	0.78	86
	3-MMC	178.12276	2.48	253
	Cholesterol	369.35176^e^	0.48	6388

^a^Yellow powder

^b^Red powder

^c^Also detected as an ammonium adduct at *m*/*z* 266.17508

^d^Target analytes are reported at 2.5 µg mL⁻^1^; diazepam was the only one also detected at 0.1 µg mL⁻^1^

^e^Value corresponding to the [M − H_2_O + H]^+^ species

As shown in Table 3, cocaine was identified as the major component in the yellow powder and MDA in the red powder, both with S/N values above 15,000 and mass errors below 7 ppm. In the seized tablet, N-MEC was detected at approximately 1% relative abundance, with an S/N of 483, indicating that the approach retained adequate detectability for low-abundance constituents, whereas MDMA yielded an S/N of 44,385, consistent with the high concentrations commonly reported for this drug in seized tablets [43, 44]. MDMA, MDA, and cocaine are among the most extensively characterized analytes in forensic science and have been widely studied in tablets, powders, and biological matrices by conventional extract-based methods using GC–MS and LC–MS/MS [35, 36, 45]. In contrast, N-MEC, a new psychoactive substance (NPS), poses a greater analytical challenge for routine protocols not specifically optimized for target analytes, having previously remained undetected in illicit tablets known to contain it when an extract-based protocol was applied in forensic practice [12].

For roasted coffee powder, the identified compounds included two characteristic alkaloids, caffeine and trigonelline, and 5-hydroxymethylfurfural (5-HMF) — a potentially carcinogenic contaminant formed during roasting. Caffeine, the most abundant constituent, yielded an S/N of 1015, whereas 5-HMF and trigonelline were detected with S/N values of 39 and 27, respectively. These analytes are well-established markers of coffee composition and processing, relevant to both quality assessment and health-related monitoring [46, 47]. They have previously been reported in DART-MS studies [13, 48], but not from roasted coffee powder under a solvent-free analytical workflow such as that employed here.

Analysis of the hair sample revealed a profile comprising endogenous lipids and biomarkers of plasticizer exposure: squalene (S/N = 1378), cholesterol detected as the dehydrated protonated species [M − H_2_O + H]^+^ (S/N = 121), commonly generated by in-source water loss under DART ionization conditions, di-N-octyl phthalate (DNOP, S/N = 2844), and mono-N-octyl phthalate (MNOP, S/N = 2543). Squalene is a major lipid of human sebum and a well-characterized surface component of untreated hair [49]. The dehydrated cholesterol species likewise reflects the typical sterol content of hair fibers. DNOP is a plasticizer widely used in PVC-based applications, including flooring, coatings, and toys, from which it can migrate into the surrounding environment, whereas MNOP is an established marker of human DNOP exposure [50, 51]. Since hair has increasingly been recognized as a suitable matrix for assessing cumulative exposure to environmental contaminants [50, 51], the simultaneous detection of both compounds is consistent with the current biomonitoring literature.

Plant material analysis revealed a chemically diverse set of sesquiterpene lactones structurally related to dehydrocostus lactone, consistent with the botanical origin of the sample, since sesquiterpene lactones are well-established bioactive secondary metabolites predominantly found within the *Asteraceae* family [52], to which wormwood belongs. Dehydrocostus lactone and structurally related sesquiterpene lactones have been characterized by extraction-based techniques — including GC–MS, LC–MS/MS, and UPLC-PDA — as target markers in quality-control and chemical fingerprinting workflows for medicinal plant materials [53, 54]. In this study, dehydrocostus lactone (*m*/*z* 231.13786, S/N = 13,668) was the major constituent detected, while several additional peaks corresponded to structurally related variants differing by dehydration, hydration, mono-oxygenation, or combinations thereof (Table 3).

Analysis of the spiked antemortem blood sample was evaluated at two concentration levels. At 0.1 µg mL⁻^1^, diazepam was the only target analyte detected, with a mass error of 0.06 ppm and an S/N of 7. At 2.5 µg mL⁻^1^, PERM-DART-HRMS enabled the simultaneous identification of multiple drug classes of forensic relevance, including benzodiazepines (diazepam and lorazepam), amphetamine-type stimulants (MDMA and MDA), a synthetic cathinone (3-MMC), and an NBOMe derivative (25I-NBOMe), alongside cholesterol, identified via its dehydrated ion ([M − H_2_O + H]^+^). At this concentration, the target drugs were detected with mass errors below 2.5 ppm and S/N ratios ranging from 4 to 381, despite the chemical and physical complexity of whole blood. Lorazepam, the lowest-intensity target drug at 2.5 µg mL⁻^1^ (S/N = 4), was consistently identified across replicates, indicating repeatable detectability even at low signal levels in a suppression-prone matrix.

These results are directly comparable to the most recent study reporting DART-HRMS analysis of spiked antemortem whole blood without sample preparation [17], in which 10 µL of blood was deposited onto a mesh screen support (OpenSpot) and analyzed for 1 min. In that study, diazepam and lorazepam were detectable only at spiked concentrations of 10 µg mL⁻^1^, above concentrations typically encountered in real overdose cases (up to approximately 5.5 µg mL⁻^1^), leading the authors to conclude that direct DART-HRMS screening of raw blood without pretreatment was not analytically feasible. In the present work, both diazepam and lorazepam were detected using half the sample volume (5 µL vs 10 µL), a shorter analysis window (48 s vs 60 s), and one-quarter of the analyte concentration previously required for detection (2.5 µg mL⁻^1^ vs 10 µg mL⁻^1^); in the case of diazepam, detection was also achieved at 0.1 µg mL⁻^1^, which represents a 100-fold lower concentration than previously reported, indicating a clear gain in analytical feasibility for this matrix under preparation-free conditions.

Overall, compound assignment was supported by low mass errors (< 5 ppm, except for MDA, which remained below 7 ppm), repeatable mass measurements across replicate analyses, expressed as the RSD of measured *m*/*z* values (< 0.001%), and S/N values compatible with the detection of ions spanning a broad abundance range, remaining above 3 even for the least intense identified analytes and exceeding 1000 for major constituents. The mass errors and RSD of measured *m*/*z* values obtained fall within the ranges reported in high-resolution MS studies that applied extract-based workflows to analogous analytes and sample types [17, 55–59]. For spiked whole blood, detection at 2.5 µg mL⁻^1^ is substantially higher than the LODs reported by LC–MS/MS for the same or similar analytes in blood (< 5 ng mL⁻^1^) [36, 37], which is expected given the absence of any enrichment or clean-up step. Importantly, the concentration levels of 2.5 and 0.1 µg mL⁻^1^ fall within concentration ranges encountered in real forensic blood samples [17, 43].

Taken together, the results show that the primary contribution of PERM-DART-HRMS lies in the operational mode through which chemically informative profiles were reliably obtained — i.e., directly from intact, unextracted samples, without solvents or sample-preparation steps, in a standardized manner with reduced operator dependence, while preserving instrument and sample integrity.

### Analytical comparison between PERM-DART-HRMS and reference DART-HRMS workflows

After evaluating the cross-matrix applicability of PERM-DART-HRMS, a focused analytical comparison was performed against traditional direct DART-HRMS (i.e., using tweezers, a spatula, or a mesh screen) and extract-based DART-HRMS. This comparison was carried out using two representative matrices: the seized tablet and the plant material. These samples were selected because the traditional direct-DART workflow could be completed for them without immediate instrumental instability, while still representing highly distinct analytical scenarios: a compact forensic sample containing drugs of abuse and a chemically complex biological plant matrix. To ensure a controlled and comparable assessment among workflows, the analytical metrics were calculated using the same initial acquisition window of 0.23 min (13.8 s) for all modes. This common window avoided favoring longer acquisitions and allowed the traditional direct-DART condition (i.e., without PERM or extraction) to be evaluated within the initial period of sample exposure, before prolonged interaction with the heated gas stream. The results are summarized in Table 4.

**Table 4:** Comparison of analytical performance among PERM-DART-HRMS, traditional direct DART-HRMS (i.e., using tweezers, a spatula, or a mesh screen), and extract-based DART-HRMS for the seized tablet and plant material. Metrics were calculated using the same initial acquisition window of 0.23 min for all workflows. S/N values are used as qualitative indicators of detectability under each workflow, not as quantitative validation parameters

Matrix	Format	Substances	*m*/*z*	Error (ppm)	S/N
Seized tablet	Raw (PERM)	MDMA	194.11730	1.37	37,731
		N-MEC	192.13842	0.65	74
	Raw (traditional)	MDMA	194.11735	1.11	57,897
		N-MEC	192.13787	2.19	236
	Extract	MDMA	194.11765	0.46	17,810
		N-MEC	n.a	n.a	n.a
Plant material	Raw (PERM)	Dehydrocostus lactone (M)	231.13790	0.28	9466
		Dehydrated dehydrocostus lactone (M – H_2_O)	213.12760	0.96	25
		Dehydrogenated dehydrocostus lactone (M + O − H_2_O)	229.12247	0.68	3791
		Hydrated dehydrocostus lactone (M + H_2_O)	249.14858	0.22	341
		Mono-oxygenated dehydrocostus lactone (M + O)	247.13299	0.47	325
		Mono-oxygenated dihydrated dehydrocostus lactone (M + O + 2H_2_O)	283.15397	0.11	8
	Raw (traditional)	Dehydrocostus lactone (M)	231.13756	1.75	7935
		Dehydrated dehydrocostus lactone (M – H_2_O)	213.12728	0.54	51
		Dehydrogenated dehydrocostus lactone (M + O − H_2_O)	229.12206	1.11	2461
		Hydrated dehydrocostus lactone (M + H_2_O)	249.14817	1.42	393
		Mono-oxygenated dehydrocostus lactone (M + O)	247.13248	1.60	720
		Mono-oxygenated dihydrated dehydrocostus lactone (M + O + 2H_2_O)	283.15281	4.20	22
	Extract	Dehydrocostus lactone (M)	231.13768	1.23	10,012
		Dehydrated dehydrocostus lactone (M – H_2_O)	213.12720	0.89	39
		Dehydrogenated dehydrocostus lactone (M + O − H_2_O)	229.12213	0.79	6022
		Hydrated dehydrocostus lactone (M + H_2_O)	249.14827	1.00	2846
		Mono-oxygenated dehydrocostus lactone (M + O)	247.13268	0.77	1000
		Mono-oxygenated dihydrated dehydrocostus lactone (M + O + 2H_2_O)	n.a	n.a	n.a

*n.a.*, not assigned/detected in the corresponding workflow

For the seized tablet, MDMA was detected in all three workflows with mass errors below 1.4 ppm and high S/N values: 37,731 under PERM mode, 57,897 under the traditional direct-DART workflow (i.e., using tweezers), and 17,810 in the extract-based workflow. N-MEC, a lower-abundance constituent of the tablet, was detected in both direct raw-sample workflows, with S/N values of 74 under PERM mode and 236 under the traditional direct-DART workflow, but was not detected in the extract-based analysis. These results show that PERM-DART-HRMS preserved the ability to detect both the major drug component and a lower-abundance constituent directly from the unextracted tablet. Although the traditional direct-DART workflow yielded intense signals, its analytical response must be interpreted together with the operational drawbacks described previously.

For the plant material, the two direct raw-sample workflows produced the same selected set of dehydrocostus lactone-related species. Under PERM-DART-HRMS, these ions were detected with mass errors between 0.11 and 0.96 ppm and S/N values ranging from 8 to 9466. Under the traditional direct workflow (i.e., using tweezers), the same ions were detected with S/N values ranging from 22 to 7935; the mass errors were slightly higher for some species, reaching up to 4.20 ppm, but remained within an acceptable range for qualitative HRMS-based assignment. The extract-based workflow yielded higher S/N values for selected ions, particularly dehydrocostus lactone, hydrated dehydrocostus lactone, and mono-oxygenated dehydrocostus lactone, with mass errors below 1.3 ppm. However, the lowest-abundance ion detected in both direct raw-sample workflows was not detected in the extract-based analysis under the evaluated conditions.

Overall, this comparison shows that the analytical value of PERM-DART-HRMS is not limited to signal generation alone. In some cases, the traditional direct workflow produced equal or higher S/N values, but this occurred under conditions associated with greater operational risk and poorer control over sample exposure. Conversely, extract-based DART-HRMS provided a useful reference point but required sample preparation and did not always reproduce the same set of ions observed in the direct raw-sample workflows. In this context, PERM-DART-HRMS preserved the broader chemical information obtained from direct analysis while avoiding the operational drawbacks associated with exposing matrices directly to the DART gas stream.

## Conclusion

A new approach — Permeably Enclosed Raw Matrices (PERM) — was developed to overcome the key operational and analytical challenges associated with the direct analysis of samples in their native state by DART-MS. By confining the matrix within a semipermeable enclosure, PERM safeguards the instrument against particulate contamination, reduces the risk of thermal damage, and eliminates operator dependence during analysis. At the same time, it renders the entire sampling and analytical process more practical and standardized. These improvements were achieved without introducing relevant new background ions from the paper enclosure or PLA/PETG 3D-printed holders and without compromising the inherent advantages of ambient ionization. The use of PERM-DART-MS therefore translates into reductions in time, labor, and chemical consumption, with direct implications for laboratory sustainability through the elimination of organic solvent use.

Applied across chemically and physically diverse matrices, PERM-DART-HRMS enabled accurate and precise detection of multiple analytically relevant constituents: secondary metabolites in plant material and coffee (terpenes and alkaloids, respectively), drugs of abuse — including NPS adulterants — in seized tablets and powders, and endogenous biomarkers together with environmental contaminants in human hair. In whole blood samples, chemically distinct psychoactive substances, ranging from benzodiazepines to amphetamine-type stimulants, were reliably detected at concentrations within ranges encountered in real forensic cases, highlighting the potential of the approach for complex and suppression-prone biological matrices. Furthermore, in comparative analyses with traditional direct and extract-based DART-HRMS, PERM-DART-HRMS preserved the broader chemical information obtained from direct untreated-sample analysis, including species not observed in the corresponding extracts.

Altogether, PERM emerges as a low-cost, preparation-free platform that, when coupled to DART-HRMS, enables rapid and reliable qualitative analysis across forensic, toxicological, and food-related applications.

## Supplementary Information

Below is the link to the electronic supplementary material.

Supplementary file1 (PDF 1.13 MB)

## Data Availability

Data will be made available on request.

## Declarations

This study involved human-derived samples, namely human hair collected from a volunteer and an antemortem blood sample donated by the Rio de Janeiro State Institute of Hematology Arthur de Siqueira Cavalcanti. The collection and use of the human hair sample were conducted in accordance with the approval granted by the Research Ethics Committee of the Faculdade de Odontologia, Universidade Federal do Rio de Janeiro (CAAE: 93056925.5.0000.0268). Informed consent was obtained from the volunteer who provided the hair sample. The use of the antemortem blood sample was approved by the Research Ethics Committee of Clementino Fraga Filho University Hospital and the Faculty of Medicine of the Federal University of Rio de Janeiro (CAAE: 56118322.4.0000.5257).
