# Supplementary material for: Permeably enclosed raw matrices direct analysis in real time mass spectrometry (PERM-DART-MS): a novel solution for the analysis of untreated samples
Source: Anal Bioanal Chem. 2026 Jul 28;418(21):6957–71. doi: 10.1007/s00216-026-06692-y (PMC13658406; doi:10.1007/s00216-026-06692-y)
Supplement: Supplementary file 1 [file 216_2026_6692_MOESM1_ESM.pdf]

SUPPLEMENTARY MATERIAL

Article title: *Permeably Enclosed Raw Matrices Direct Analysis in Real Time Mass Spectrometry (PERM-DART-MS): A novel solution for the analysis of untreated samples*

Authors: Alexandre Narcelli Pestana de Aguiar [a]* (ORCID: 0009-0003-5780-3904), Luis Miguel Gutiérrez Beleño [a] (ORCID: 0000-0003-3704-5838), Francisco Radler de Aquino Neto [a] (ORCID: 0000-0003-2374-9228), Gabriela Vanini (ORCID: 0000-0002-0501-8336) [a].

[a] *Universidade Federal do Rio de Janeiro, Instituto de Química, NAF – LADETEC, Rio de Janeiro, RJ 21941-909, Brazil.*

**corresponding author: alexandrenarcelli@gmail.com*

Table S1. Chemical structures and theoretical *m/z* values of the analytes identified in the analysed samples.

Compound	Structure	Formula	Theoretical *m/z* [M+H]$^+$
MDMA	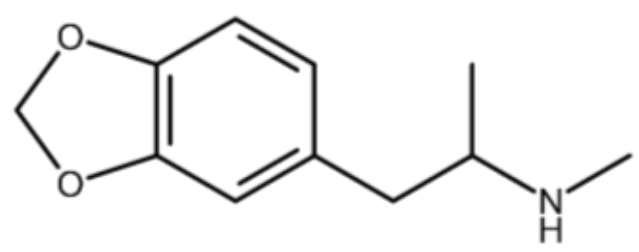	$C_{11}H_{15}NO_2$	194.11756
N-MEC	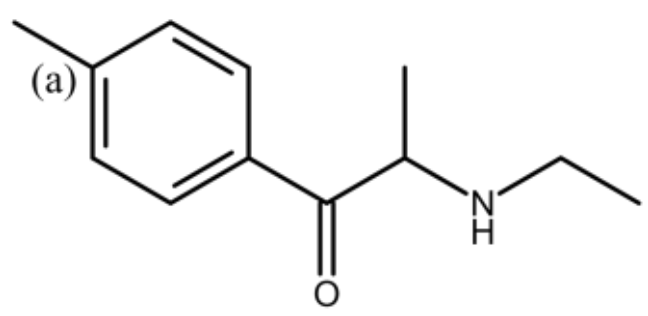<br>(a) positional isomer not specified	$C_{12}H_{17}NO$	192.13829

Cocaine	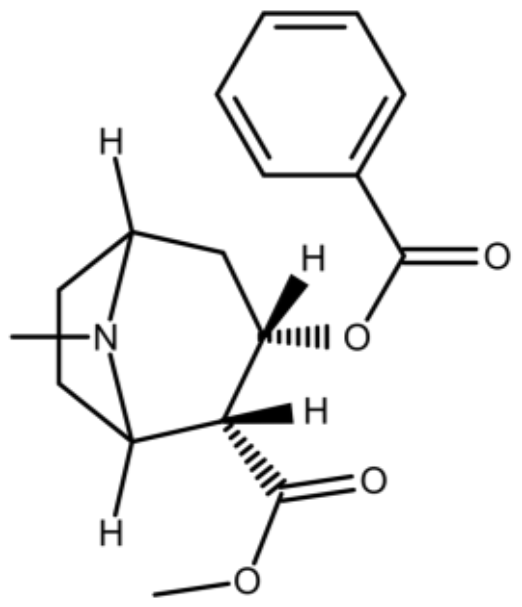	$C_{17}H_{21}NO_4$	304.15433
MDA	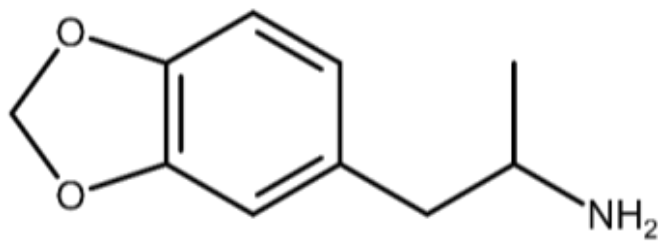	$C_{10}H_{13}NO_2$	180.10191
Caffeine	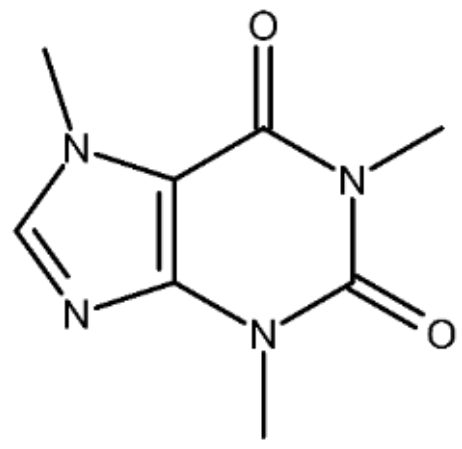	$C_8H_{10}N_4O_2$	195.08765
5-HMF	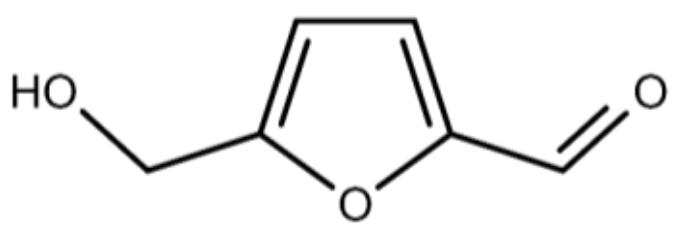	$C_6H_6O_3$	127.03952
Trigonelline	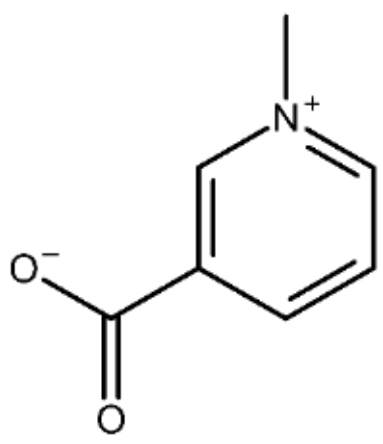	$C_7H_7NO_2$	138.05495
Squalene	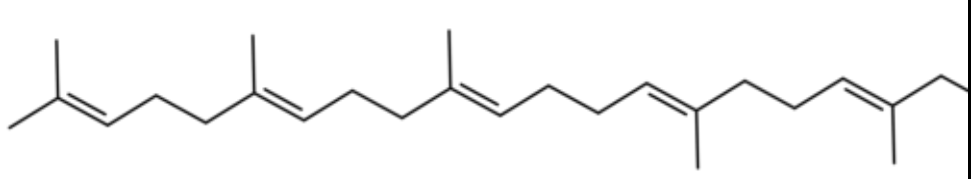	$C_{30}H_{50}$	411.39853
Cholesterol	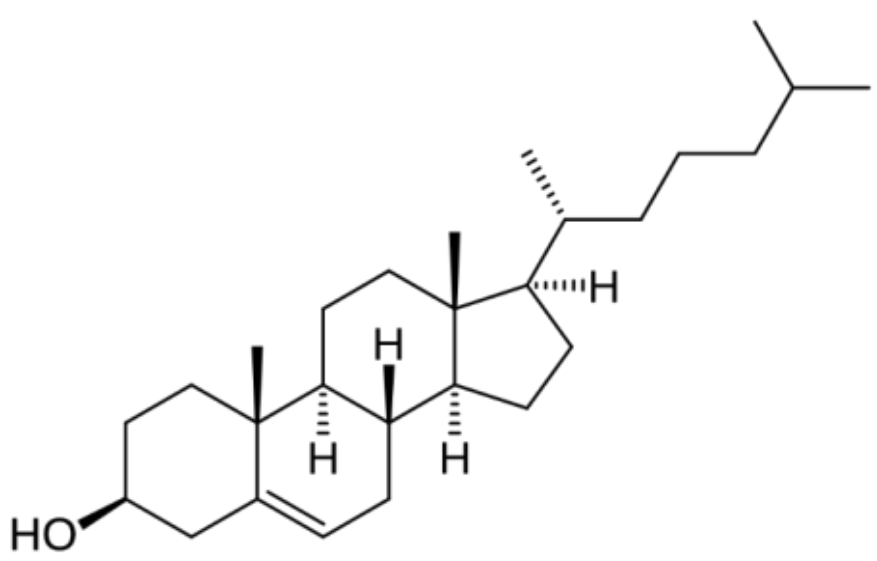	$C_{27}H_{46}O$	369.35158*

Di-N-octyl phthalate	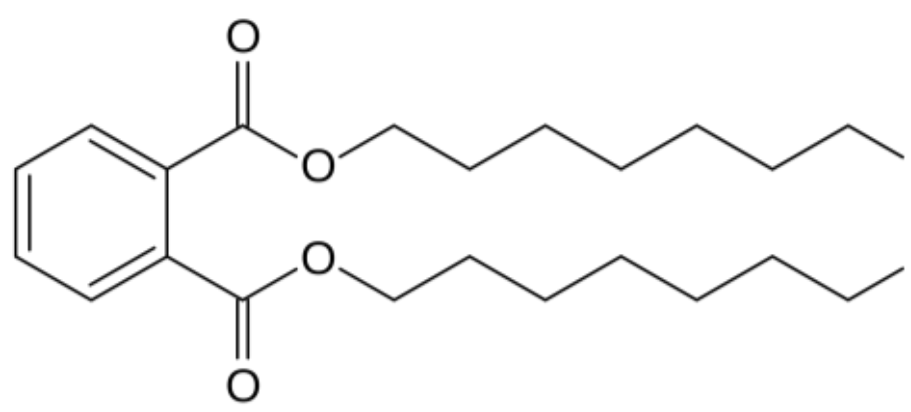	$C_{24}H_{38}O_4$	391.28429
Mono-N-octyl phthalate	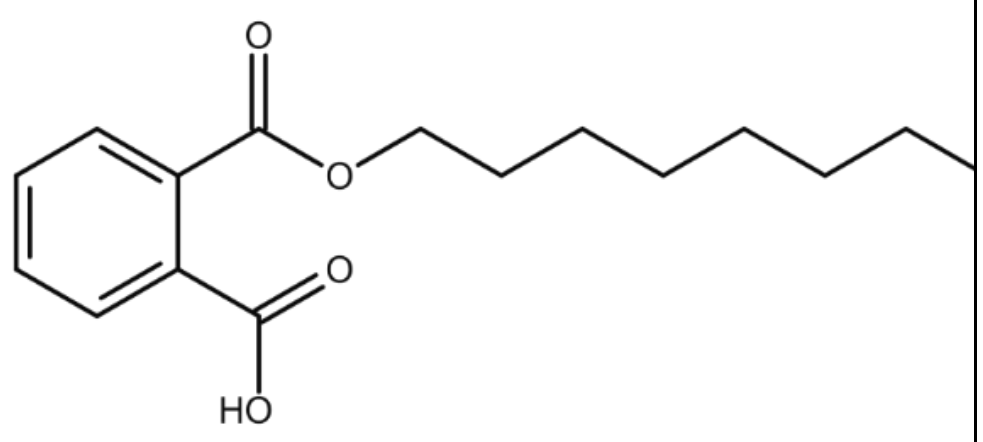	$C_{16}H_{22}O_4$	279.15909
Dehydrocostus lactone	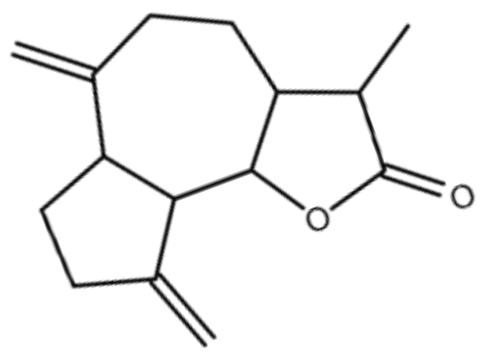	$C_{15}H_{18}O_2$	231.13796
Diazepam	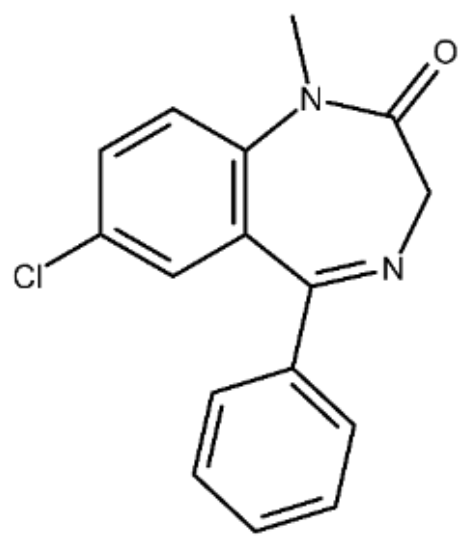	$C_{16}H_{13}ClN_2O$	285.07892
Lorazepam	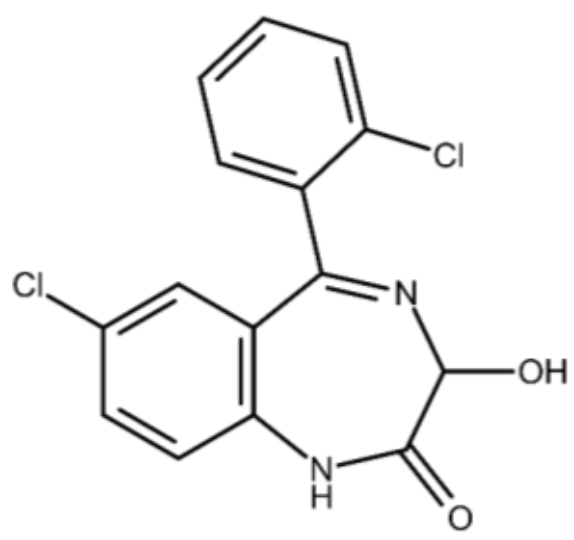	$C_{15}H_{10}Cl_2N_2O_2$	321.01921
25I-NBOMe	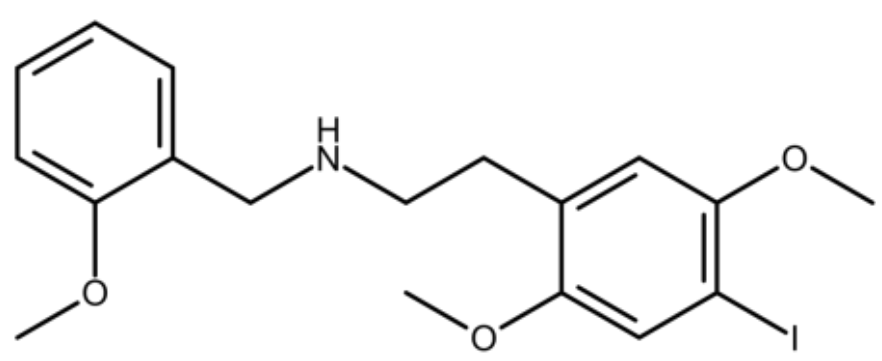	$C_{18}H_{22}INO_3$	428.07172
3-MMC	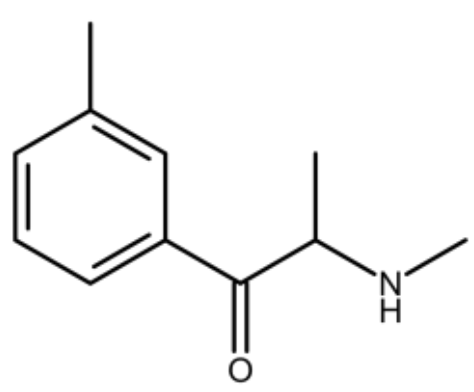	$C_{11}H_{15}NO$	178.12264

*Value corresponding to the $[M - H_2O + H]^+$ species.

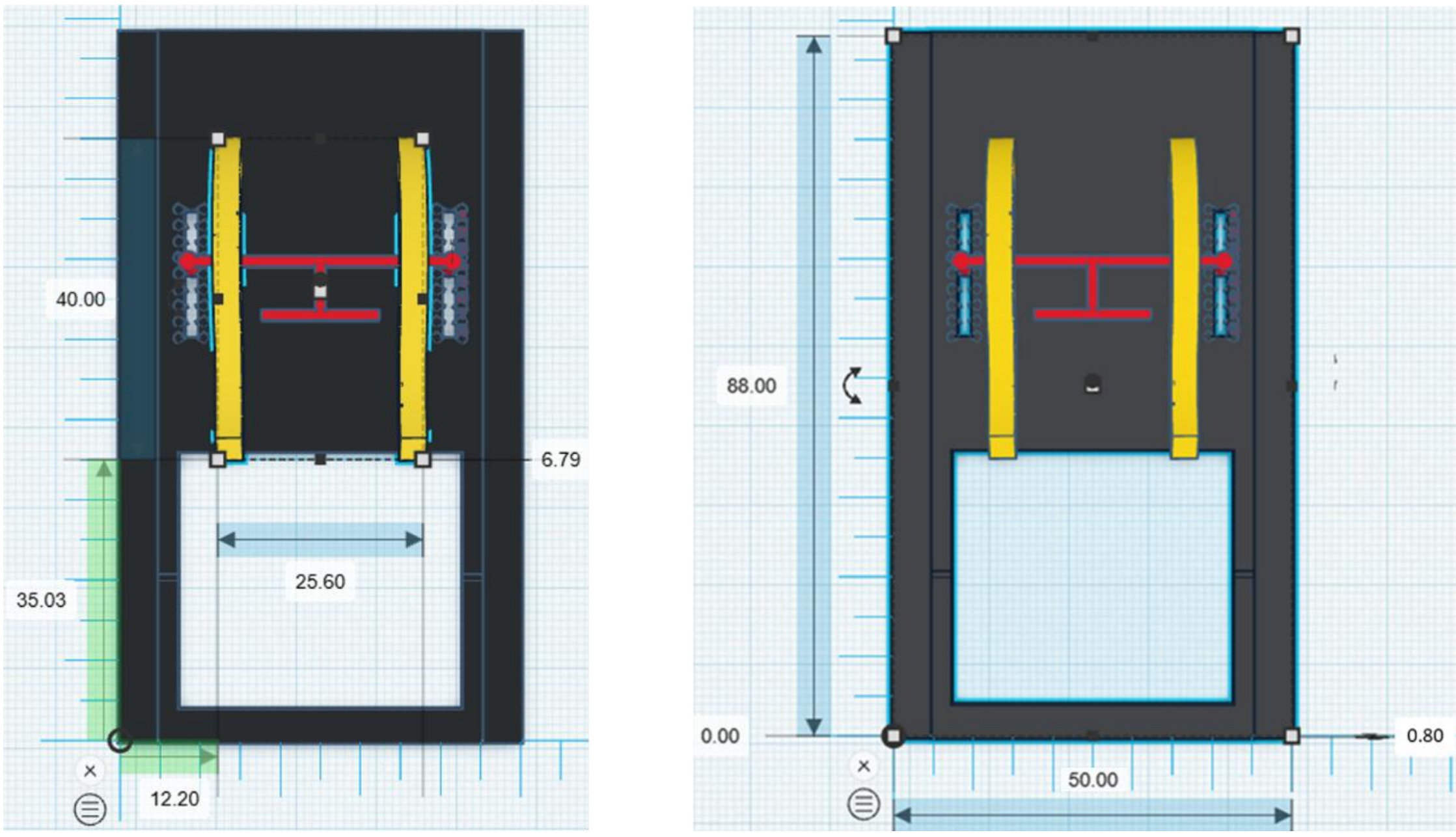

Fig. S1. 3D-printing design of the holder used for positioning the folded enclosure in the DART-MS ionization region. The scheme shows the main dimensions of the device, including the pressure arms for enclosure fixation, the height-adjustable marker used to define the insertion level, and the open rectangular window aligned with the DART source outlet and MS inlet.

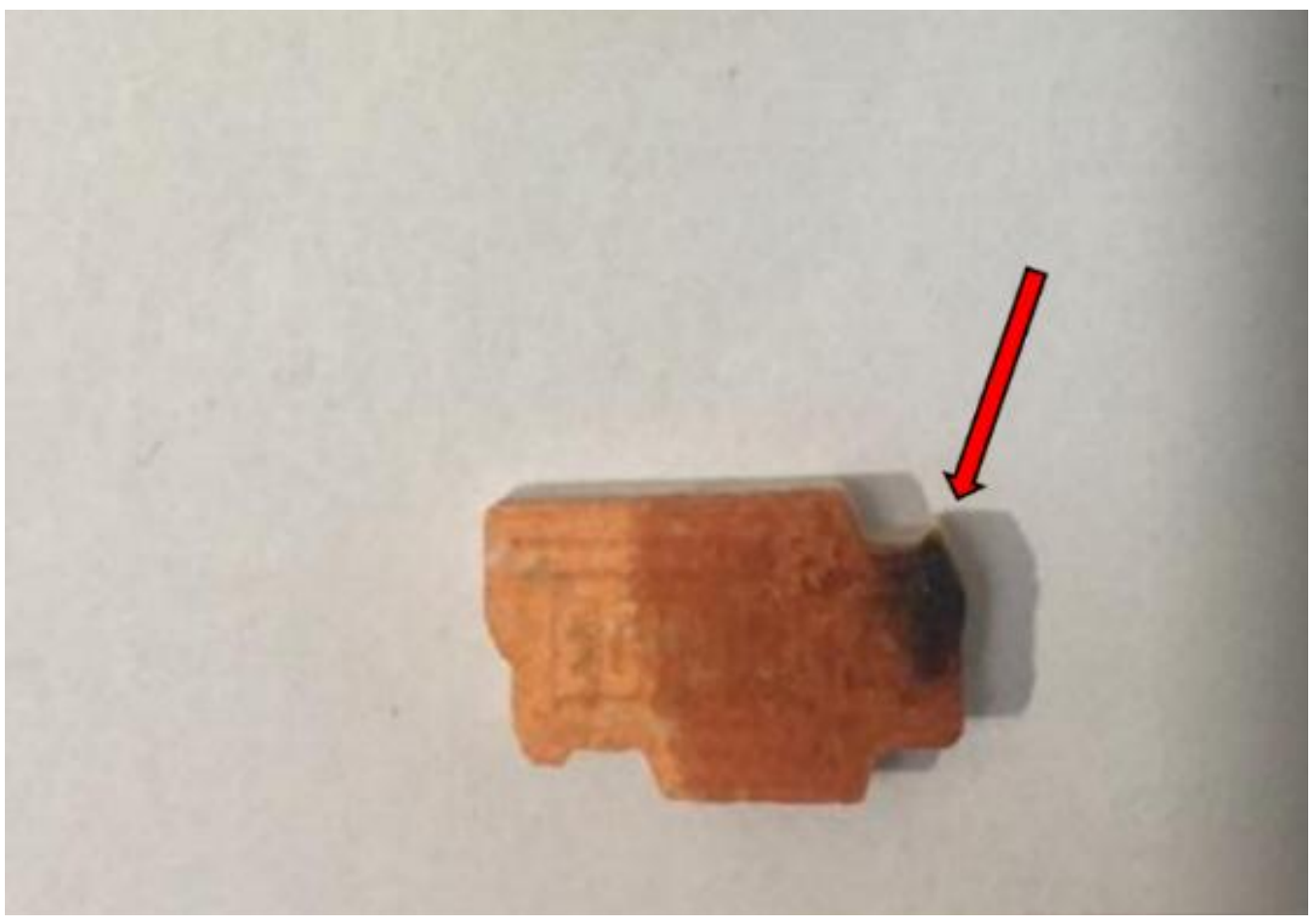

Fig. S2. Thermal damage observed on the seized tablet surface after direct exposure to the DART-HRMS ionization region without PERM.

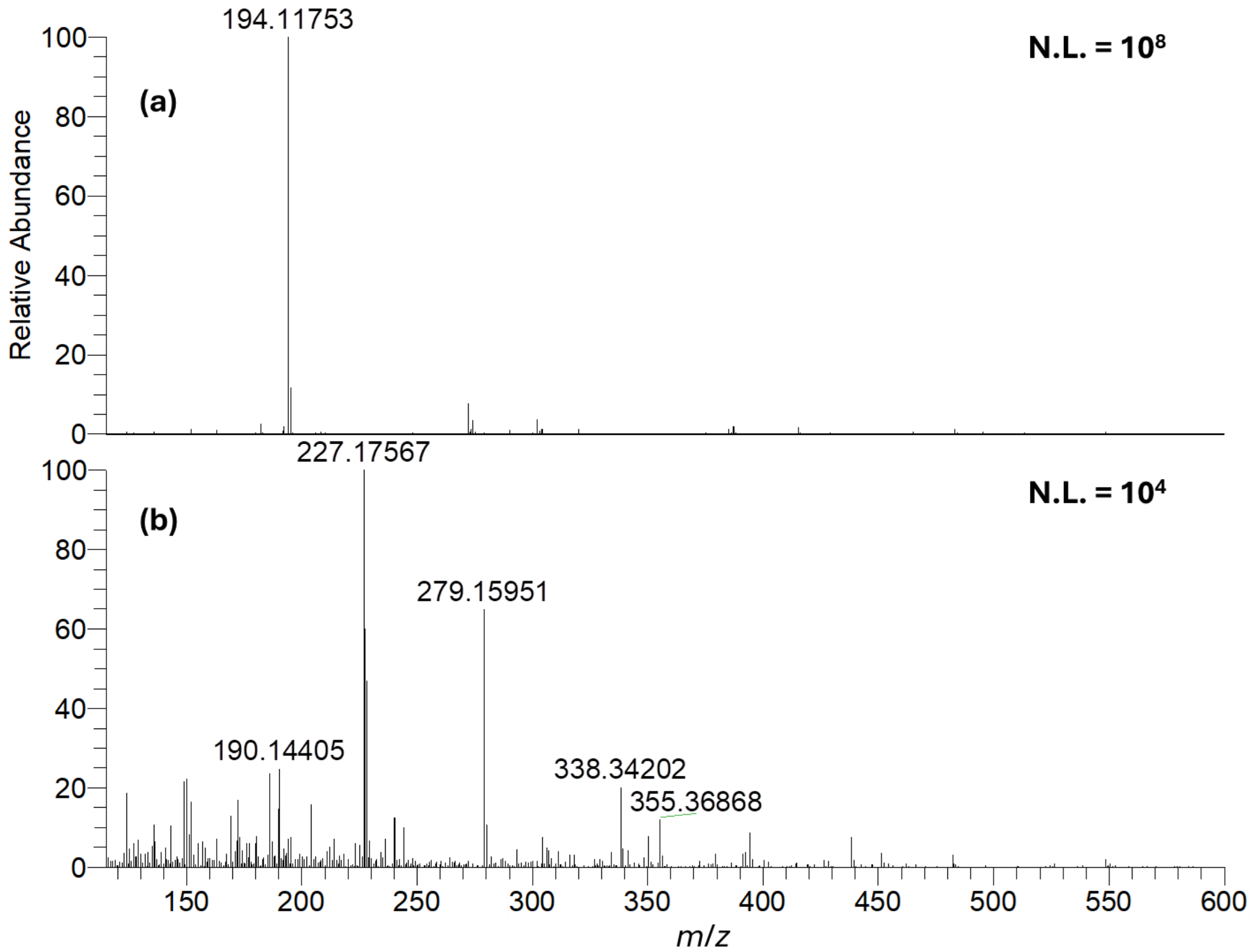

Fig. S3. Blank spectra acquired after direct tablet analysis using tweezers (a) and with PERM (b), showing intense MDMA (m/z 194.11753) carryover associated with the traditional approach. Normalized Level (N.L.) values, corresponding to signal intensity, are indicated in the spectra.

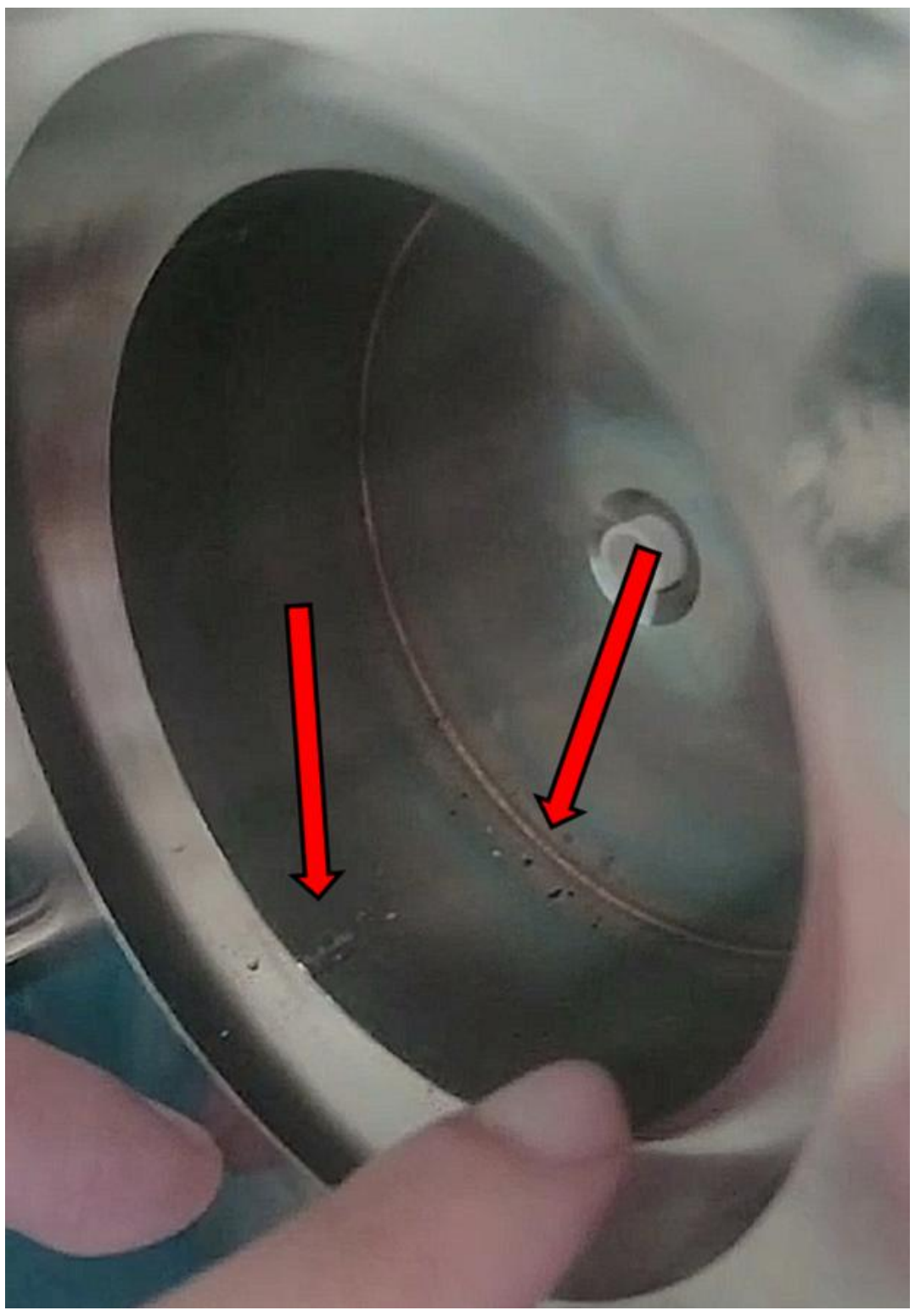

Fig. S4. Particulate contamination of the DART-HRMS vapour interface after direct seized powders and tablet analysis (*i.e.*, without PERM).

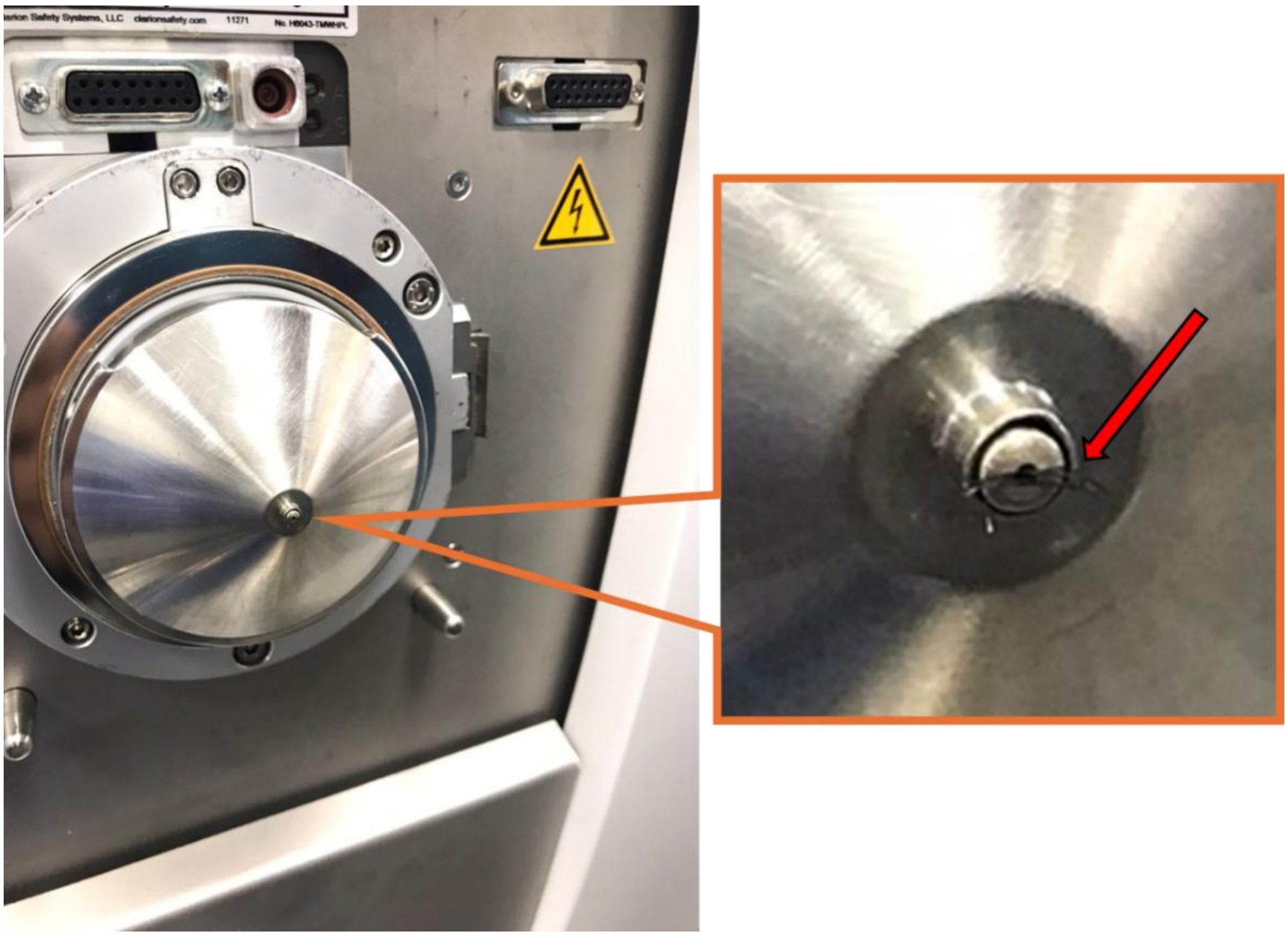

Fig. S5. Hair strand obstructing the MS inlet after hair analysis under the traditional approach (*i.e.*, without PERM).

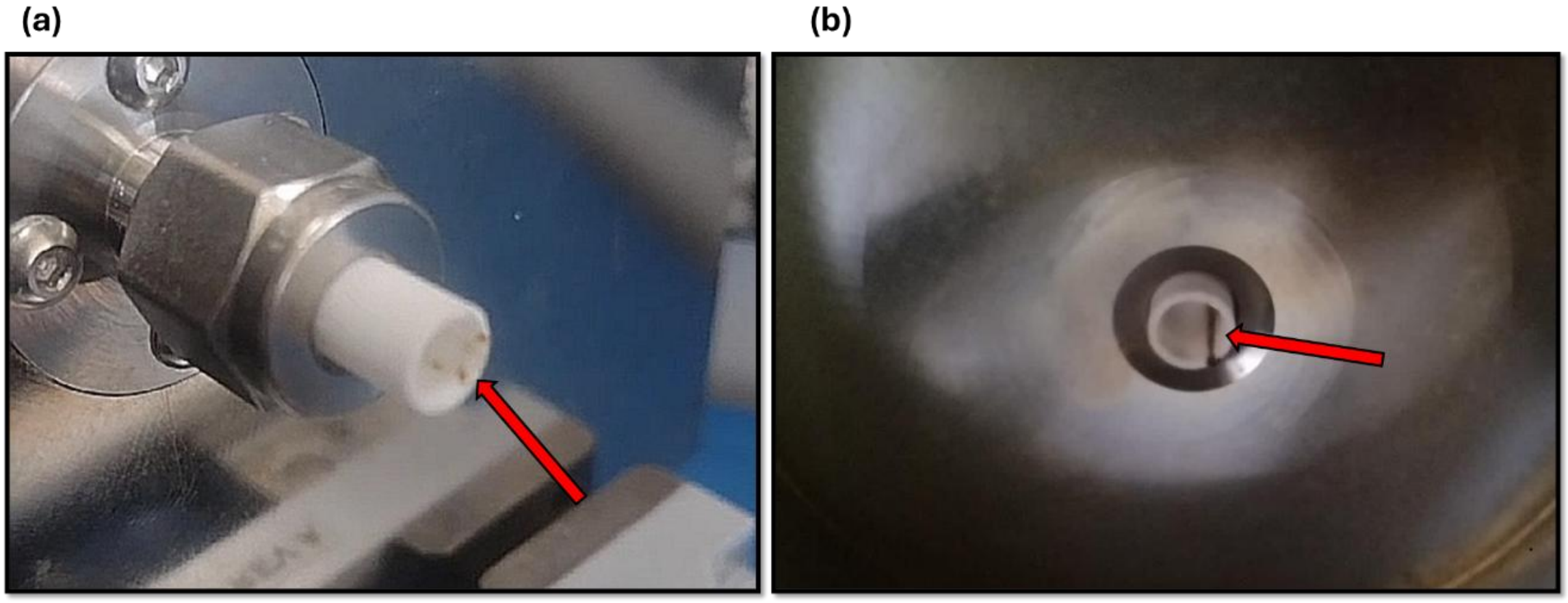

Fig. S6. Porcelain MS inlet component showing blood contamination after direct whole-blood analysis using the traditional approach (*i.e.*, without PERM): front surface (a) and inner region (b).

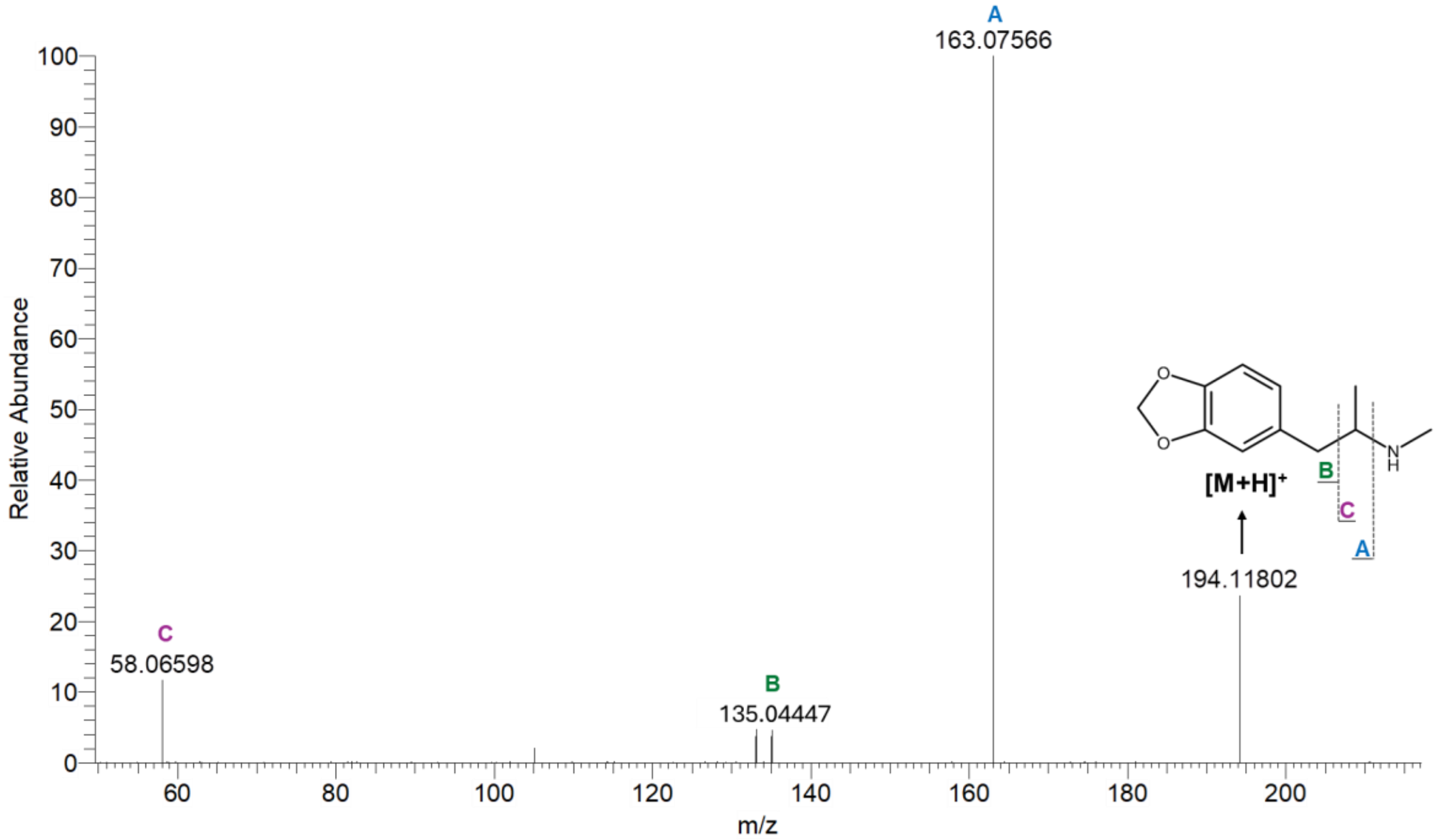

Fig. S7. MS² spectrum of MDMA obtained during PERM-DART-HRMS analysis of a seized tablet.

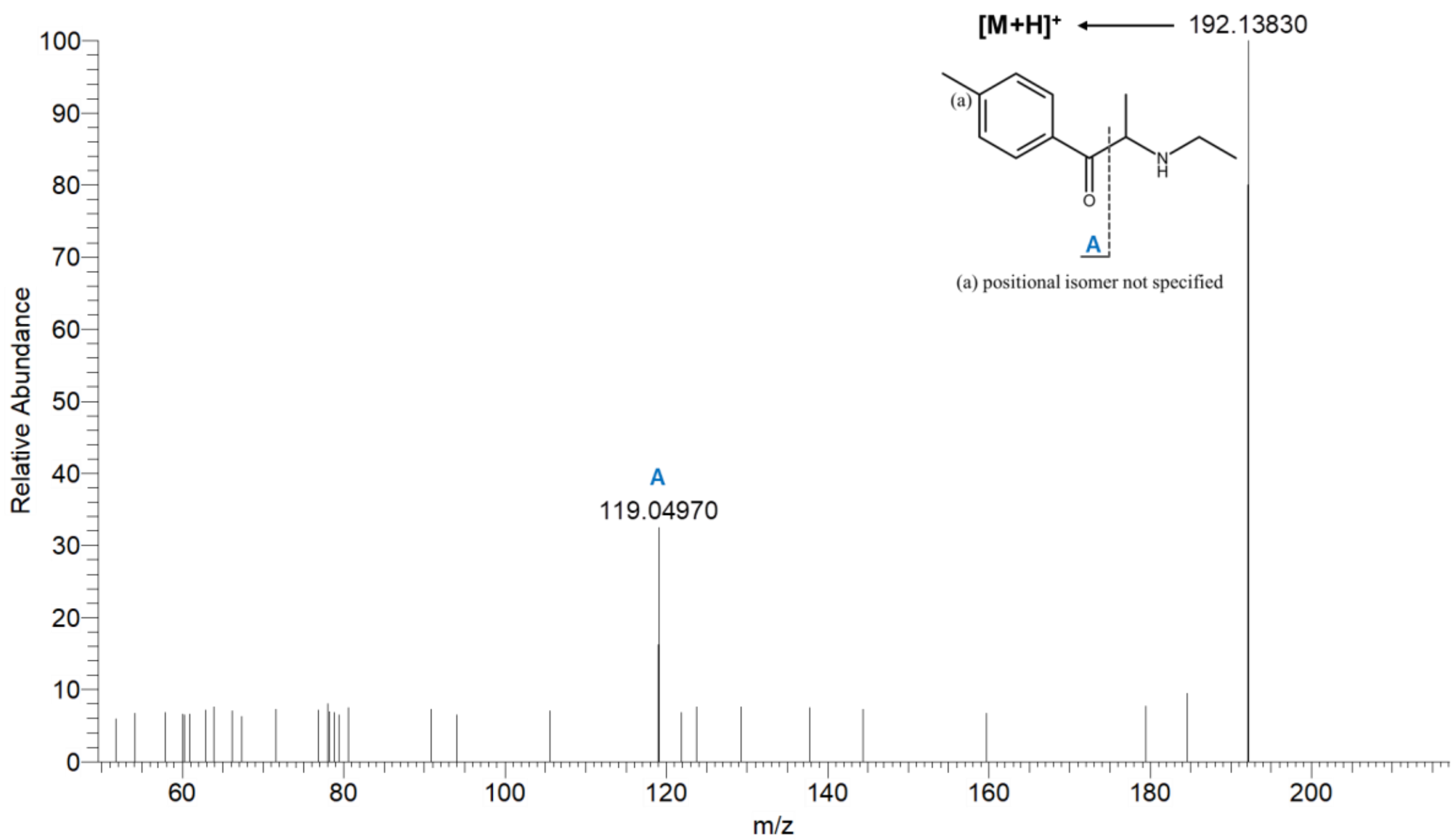

Fig. S8. MS² spectrum of N-MEC obtained during PERM-DART-HRMS analysis of a seized tablet.

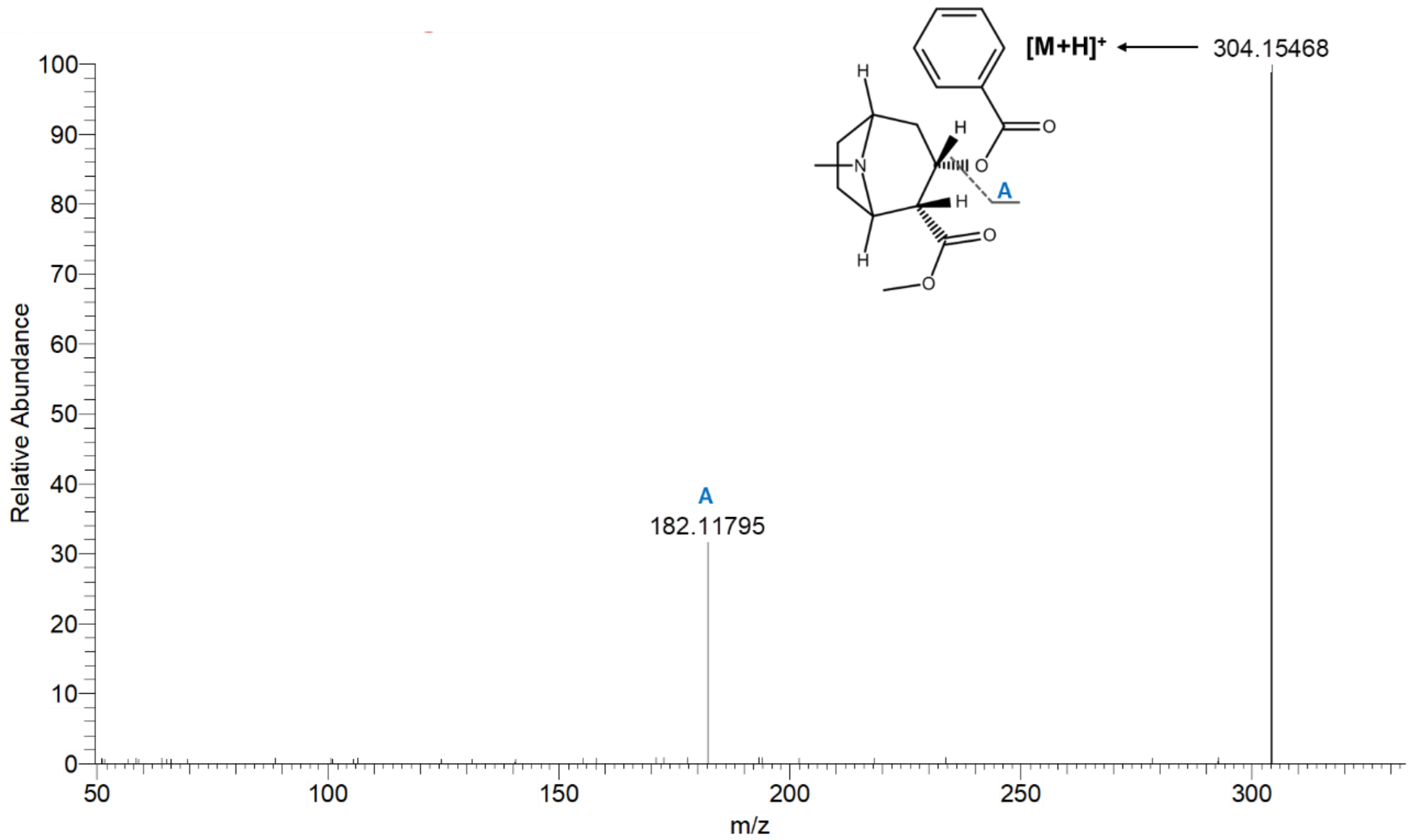

Fig. S9. MS² spectrum of cocaine obtained during PERM-DART-HRMS analysis of the yellow seized powder.

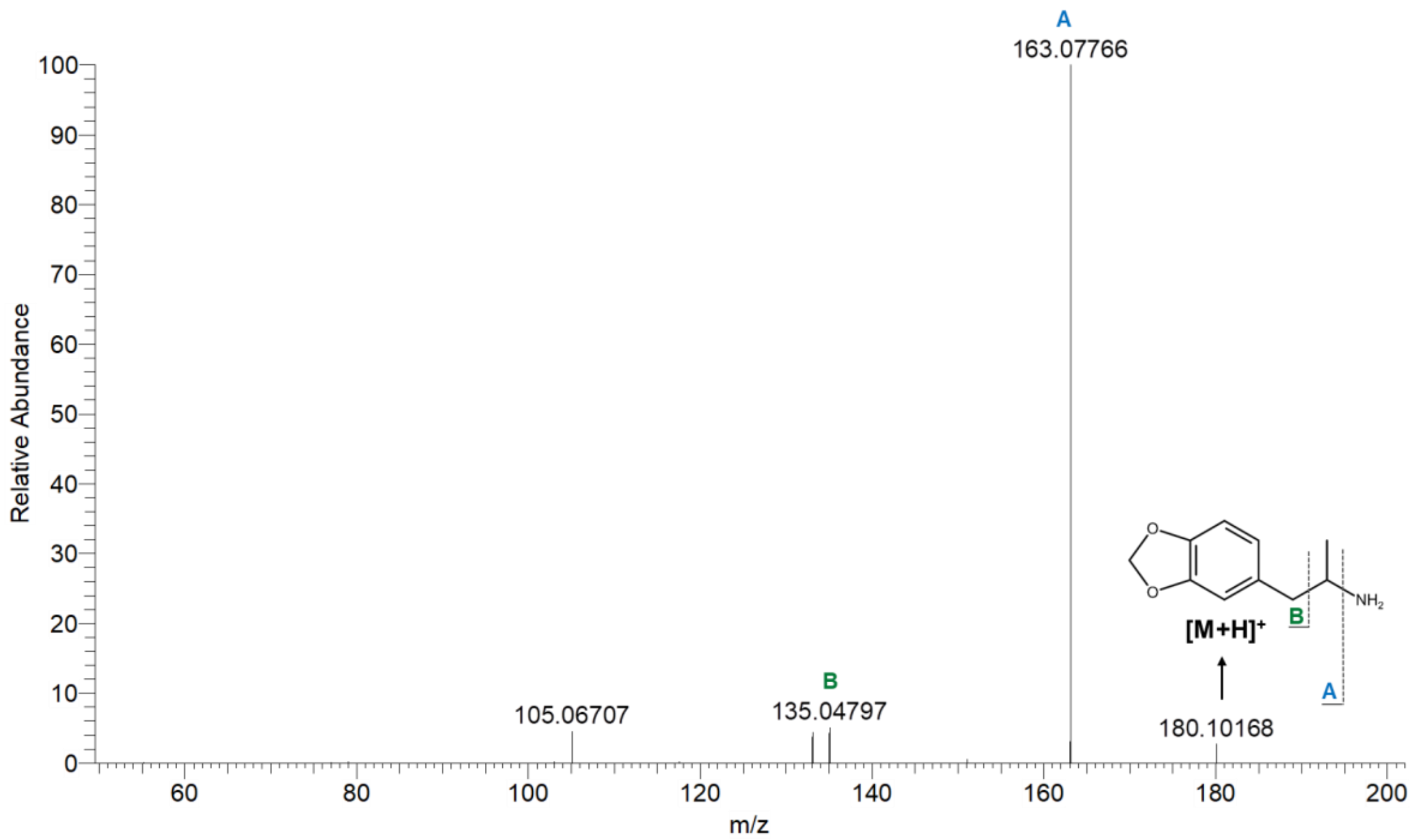

Fig. S10. MS² spectrum of MDA obtained during PERM-DART-HRMS analysis of the red seized powder.

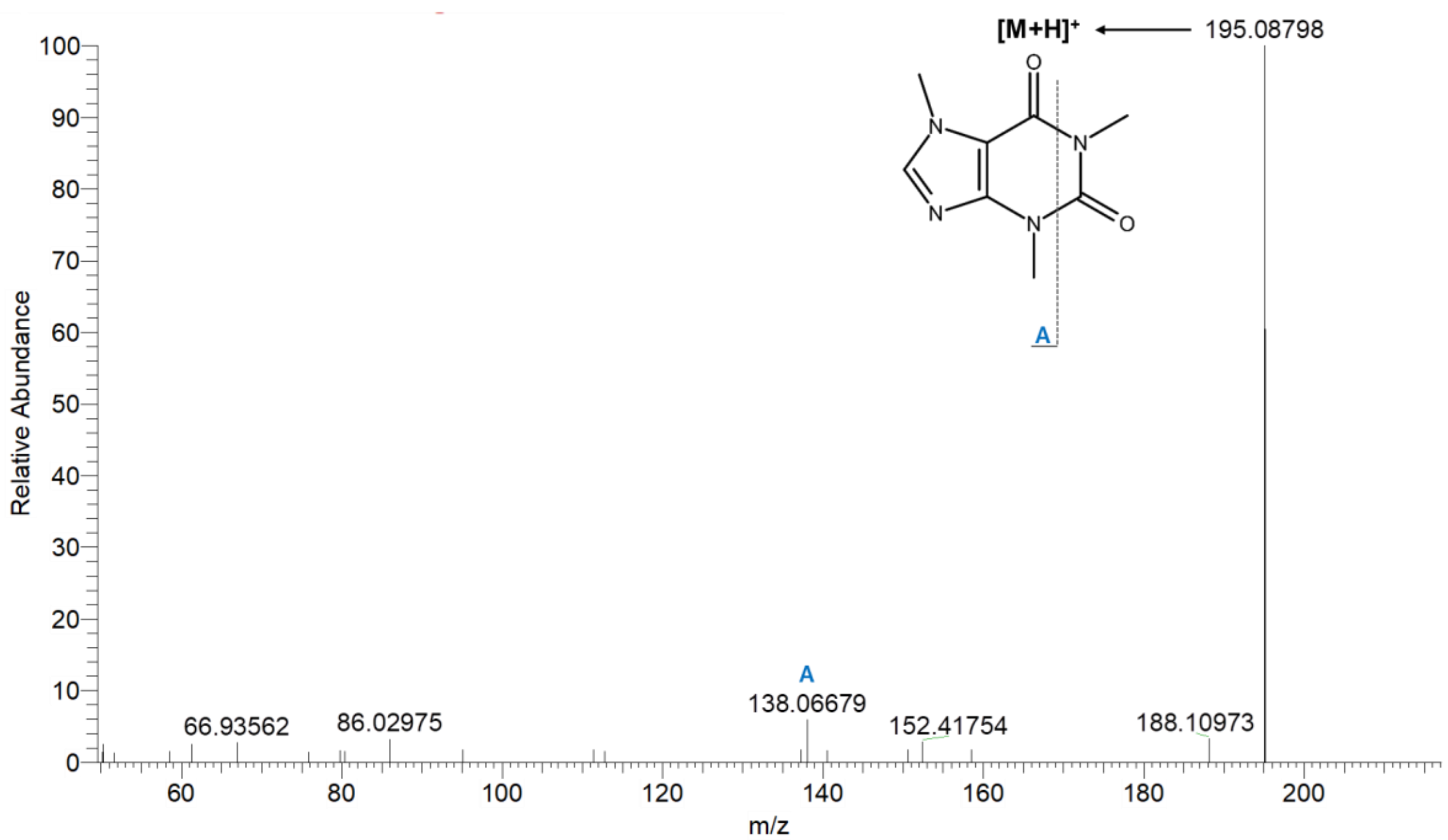

Fig. S11. MS² spectrum of caffeine obtained during PERM-DART-HRMS analysis of roasted coffee powder.

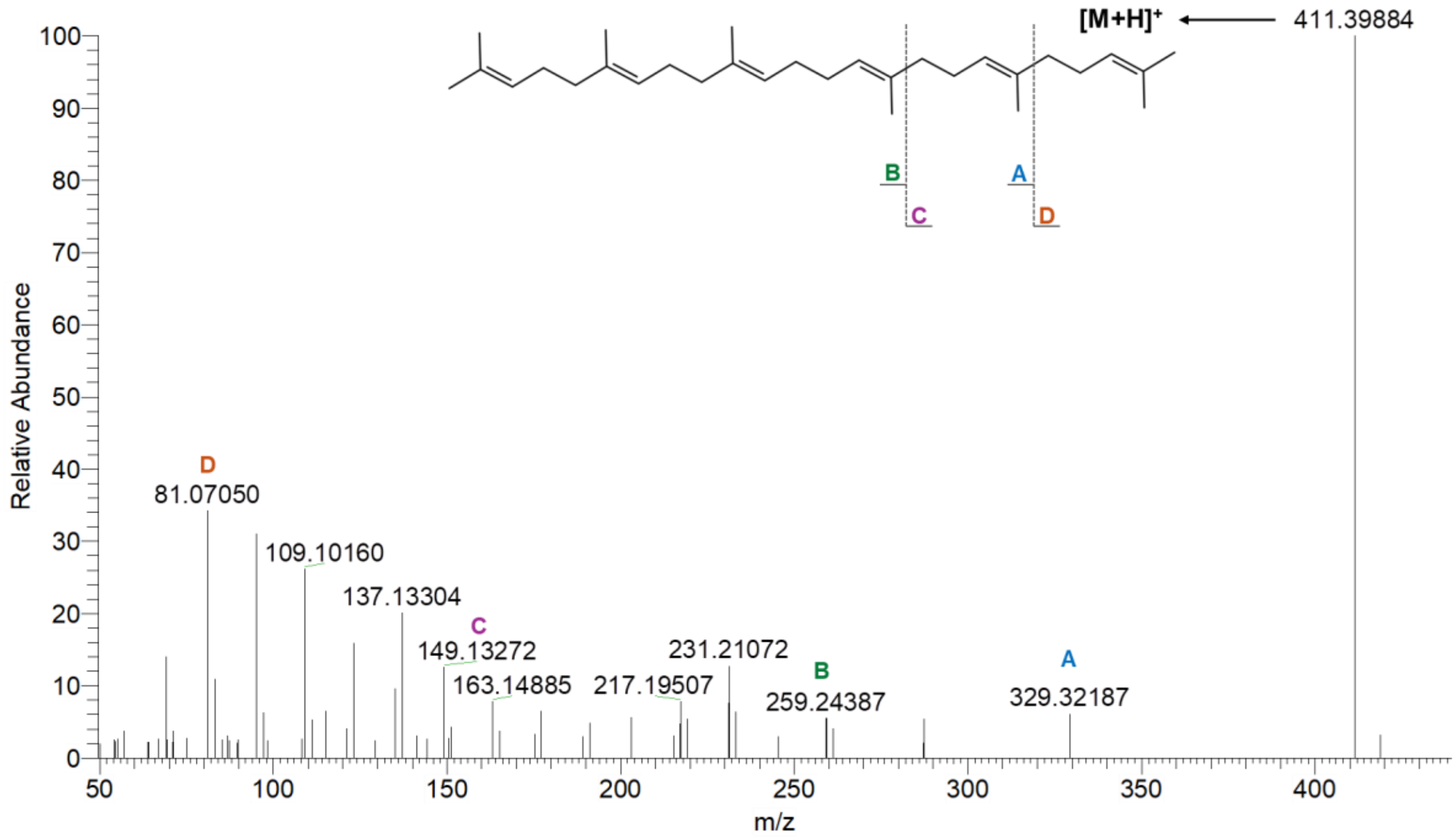

Fig. S12. MS² spectrum of squalene obtained during PERM-DART-HRMS analysis of human hair.

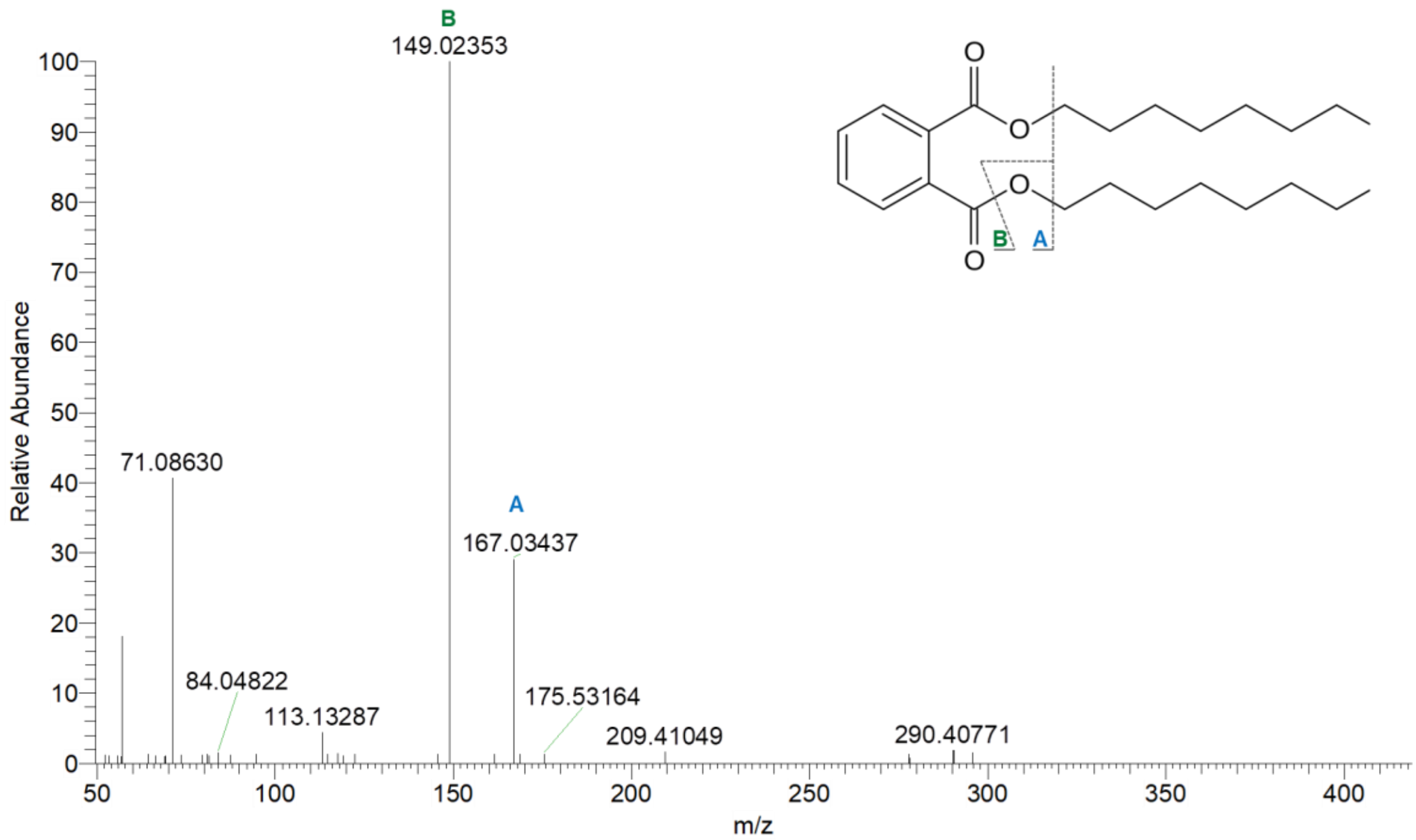

Fig. S13. MS² spectrum of di-N-octyl phthalate obtained during PERM-DART-HRMS analysis of human hair.

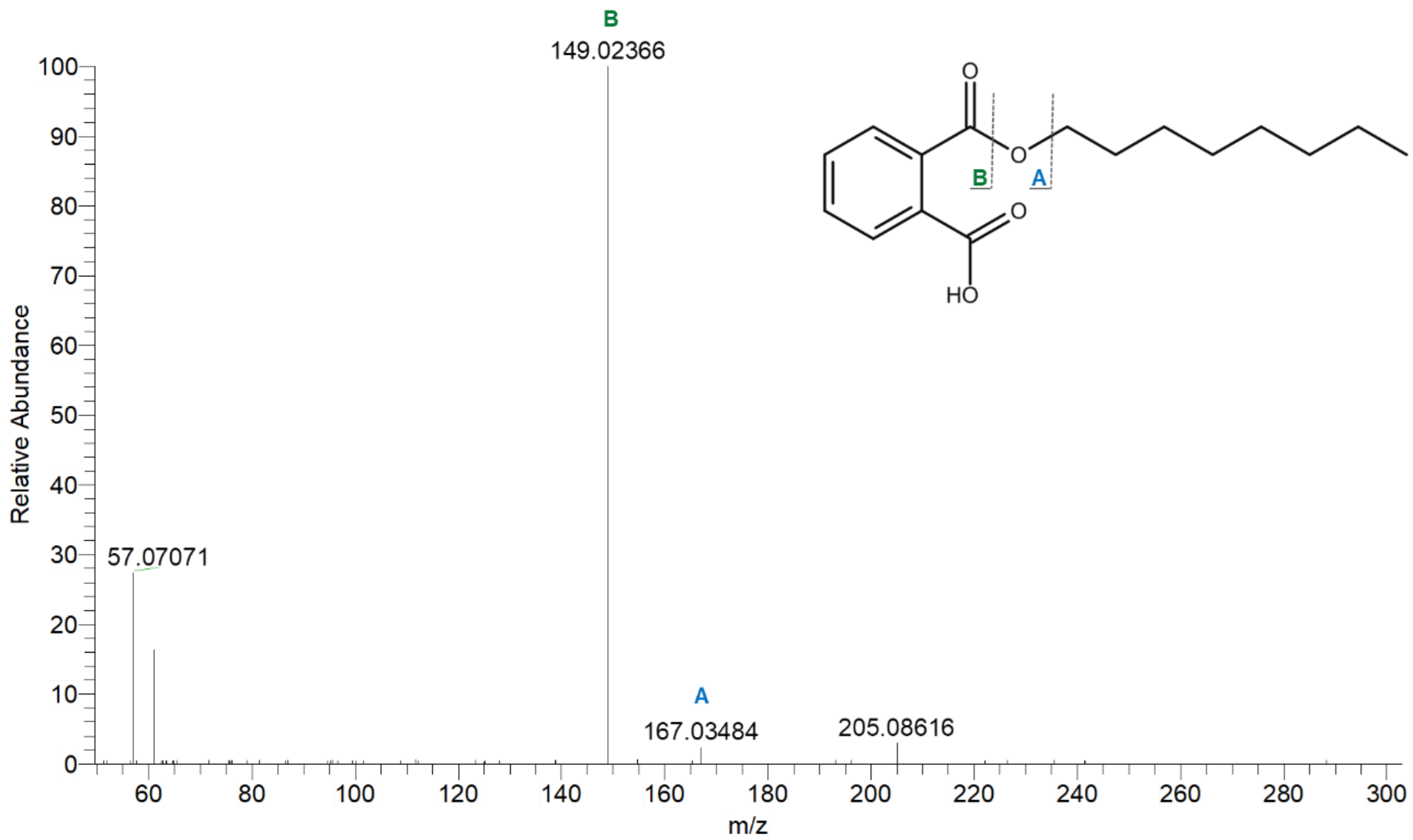

Fig. S14. MS² spectrum of mono-N-octyl phthalate obtained during PERM-DART-HRMS analysis of human hair.

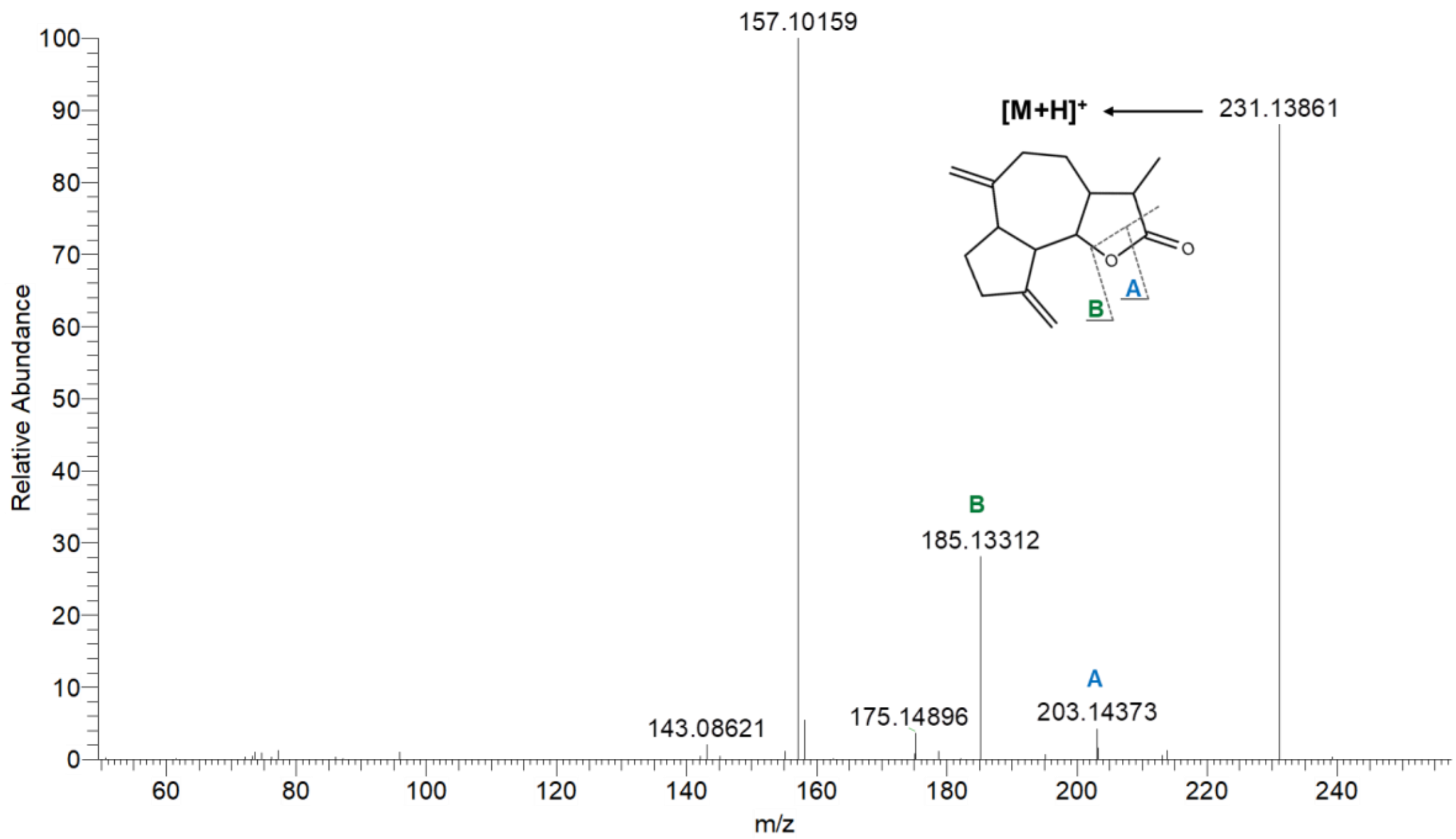

Fig. S15. MS² spectrum of dehydrocostus lactone obtained during PERM-DART-HRMS analysis of plant material.
